# Autistic traits in childhood and post‐traumatic stress disorder as young adults: a cohort study

**DOI:** 10.1111/jcpp.14163

**Published:** 2025-03-25

**Authors:** Alice M.G. Quinton, Freya Rumball, Angelica Ronald, Helen L. Fisher, Louise Arseneault, Francesca Happé, Andrea Danese

**Affiliations:** ^1^ Social, Genetic & Developmental Psychiatry Centre Institute of Psychiatry, Psychology and Neuroscience, King's College London London UK; ^2^ Adult Autism Assessment Service Oxleas NHS Foundation Trust Bexley UK; ^3^ School of Psychology Faculty of Health and Medical Sciences, University of Surrey Guildford UK; ^4^ ESRC Centre for Society and Mental Health, King's College London London UK; ^5^ Department of Child & Adolescent Psychiatry Institute of Psychiatry, Psychology and Neuroscience, King's College London London UK; ^6^ National and Specialist CAMHS Trauma and Anxiety Clinic South London and Maudsley NHS Foundation Trust London UK

**Keywords:** Autism, autism spectrum, trauma, PTSD, psychopathology

## Abstract

**Background:**

Despite the higher prevalence of childhood traumatic experiences and post‐traumatic stress disorder (PTSD) in autistic adults, research on trauma‐related psychopathology and autistic traits in young people is lacking. This study examined if high autistic traits in childhood predispose individuals to traumatic experiences, the development of PTSD and general psychopathology, and greater functional impairment by age 18, in both the general population and a subsample of trauma‐exposed young people.

**Methods:**

Data were utilised from the Environmental Risk (E‐Risk) Longitudinal Twin Study, a nationally representative cohort of 2,232 same‐sex twins born in 1994–1995 across England and Wales. Participants were a subset of children whose parents completed the Childhood Autism Spectrum Test (CAST), during assessments at ages 8, 9 and/or 12 years (*N* = 1,504). We tested associations between autistic traits in childhood and age‐18 reports of lifetime trauma exposure, lifetime PTSD diagnosis, general psychopathology (‘*p*‐factor’) and NEET status (‘not in employment, education or training’). Analyses were conducted controlling for sex, family socioeconomic status (SES), intelligence quotient (IQ) and accounting for family clustering.

**Results:**

Higher autistic traits in childhood were significantly associated with greater reports of lifetime trauma exposure (Odd Ratio [OR] = 1.26, 95% Confidence Intervals [CI] = 1.03; 1.54), lifetime PTSD diagnosis (OR = 1.91, 95% CI = 1.29; 2.82), general psychopathology (beta = 3.22, 95% CI = 1.84; 4.60) and NEET status (OR = 1.48, 95% CI = 1.05; 2.09) at age 18. Only the associations of autistic traits with PTSD and general psychopathology were robust to adjustment for potential confounders. Among trauma‐exposed children, autistic traits were also significantly associated with lifetime PTSD diagnosis (OR = 1.75, 95% CI = 1.15; 2.68) and psychopathology (beta = 3.36, 95% CI = 0.68; 6.04) at age 18, but only the association with PTSD held when adjusted for confounders.

**Conclusions:**

Our findings suggest a need to develop targeted assessments and evidence‐based treatments for PTSD to meet the needs of children with high autistic traits. However, whether our findings extend to diagnosed autistic children requires further investigation.

## Background

Post‐traumatic stress disorder (PTSD) is a debilitating psychiatric condition that can develop after experiencing a traumatic event (Danese, McLaughlin, Samara, & Stover, 2020). PTSD is characterised by persistent re‐experiencing of traumatic events through intrusive and distressing memories, as well as negative appraisals, avoidance and hypervigilance to threat. In this article, we examine if higher autistic traits in childhood are associated with trauma exposure, PTSD and broader psychopathology and functional impairment in youths. Clinical, cognitive and genetic findings in the literature suggest that this might be the case.

Compared with non‐autistic peers, autistic children have a greater risk of adversities, such as bullying and victimisation (Hoover & Kaufman, 2018). Autistic children also show a higher prevalence of mental health conditions than their non‐autistic peers (Kerns, Rast, & Shattuck, 2020) and might be at a greater risk of developing PTSD and other types of psychopathology after trauma exposure (Kerns, Newschaffer, & Berkowitz, 2015). Research with adults suggests that autistic traits may facilitate the development of trauma‐related psychopathology. In a large study, 251 middle‐aged and older adults (aged 50–81) with high autistic traits showed a 12‐fold increase in passing the cut‐off for probable PTSD when compared with 9,179 age‐ and gender‐matched low‐trait controls (Stewart et al., 2020). Likewise, a study in 103 typically developing adults aged 18–34 showed positive associations between autistic traits and PTSD symptoms, particularly hyper‐arousal symptoms (Haruvi‐Lamdan, Lebendiger, Golan, & Horesh, 2019). However, empirical research on trauma‐related psychopathology in children and young people on the autistic spectrum is lacking.

Children with higher autistic traits likely share similar cognitive styles to autistic children (Best, Moffat, Power, Owens, & Johnstone, 2008). Rumball, Happé, & Grey (2020)have suggested that specific cognitive characteristics of autism may impact how a negative or traumatic event is processed and perceived and may predispose individuals to the development of PTSD symptoms. Popular models of PTSD highlight the role of cognitive factors in promoting the development and maintenance of PTSD symptoms (Brewin, Dalgleish, & Joseph, 1996; Ehlers & Clark, 2000). Research has shown that several of these cognitive risk factors are commonly found in autistic people or those with more autistic traits, including detail‐focused processing (Happé & Frith, 2006), sensory sensitivities (Weiland, Polderman, Hoekstra, Smit, & Begeer, 2020), rumination (Golan, Haruvi‐Lamdan, Laor, & Horesh, 2022), emotional dysregulation (Mazefsky, Borue, Day, & Minshew, 2014), social withdrawal (Brosnan & Gavin, 2023), and poor verbal working memory (Wang et al., 2017). Studies with autistic adults have also reported that specific cognitive features common to ASD relate to increased PTSD symptoms, such as everyday and working memory deficits (Rumball, Brook, Happé, & Karl, 2021), brooding rumination (Golan et al., 2022) and thought suppression (Rumball, Antal, et al.,  2021).

Genetic research also suggests possible links between high autistic traits and risk for traumatic experiences. Traumatic experiences are partly influenced by heritable factors (Dahoun et al., 2024). Previous research showed that higher polygenic scores for autism may be associated with self‐reported childhood trauma (Peel et al., 2022; Ratanatharathorn et al., 2021; Warrier & Baron‐Cohen, 2021; but see Sallis et al., 2021 for contrary results). However, it is unclear if polygenic scores for autism also increase the risk of trauma‐related psychopathology (Huckins et al., 2021). Genetic research has also highlighted that similar genetic factors influence diagnosed autism and autistic traits in the general population (Robinson et al., 2011). Along with consistent behavioural and genetic evidence (Happé and Frith, 2020), these findings support a dimensional approach to examining the role of autistic traits in trauma‐related psychopathology and related functional impairment. Although high autistic traits clearly do not equate to an autism diagnosis, research on autistic traits can provide novel insights into liability to trauma exposure and trauma‐related psychopathology in the general population and prompt more focused work in clinical samples.

To examine the wider impact of trauma beyond the risk for PTSD and general psychopathology, it is also important to test whether children with high autistic traits have greater functional impairment in daily life compared with peers with less autistic traits. Being ‘not in education or employment’ (NEET) is a common metric of functional impairment in young adults. Previous work in a large, longitudinal UK‐based study found that young people in the general population with a history of trauma were more likely to be NEET than unexposed peers, and those with PTSD were more likely to be NEET than those without PTSD (Lewis et al., 2019). However, it is unclear how the presence of both trauma exposure and high autistic traits could affect this measure of functional impairment.

Building on the evidence above, in this study, we investigated if higher autistic traits in childhood predispose individuals to trauma exposure, PTSD and worse general psychopathology, as well as greater functional impairment by age 18 in a large UK birth cohort. We considered that any associations between autistic traits and trauma‐related psychopathology and functional impairment could either stem from a greater likelihood of developing negative outcomes after trauma exposure or reflect a greater likelihood of being exposed to trauma. To disentangle the relative contributions of the two mechanisms, we therefore repeated the analyses in a subset of the overall sample, including only trauma‐exposed young people.

## Method

This study was pre‐registered with OSF (https://osf.io/uf9t7/).

### Sample

Participants were members of the Environmental Risk (E‐Risk) Longitudinal Twin Study, which tracks the development of 2,232 British children. The sample was drawn from a larger birth cohort of twins born in England and Wales in 1994–1995, the Twins Early Development Study (TEDS) (Trouton, Spinath, & Plomin, 2002). Full details about the sample are reported in Appendix S1 and described elsewhere (Moffitt & the E‐Risk Study Team, 2002). Briefly, E‐Risk was constructed in 1999–2000, when 1,116 families (93% of those eligible) with same‐sex 5‐year‐old twins participated in home‐visit assessments. This sample comprised 56% monozygotic and 44% dizygotic twin pairs; sex was evenly distributed within zygosity (49% male); 90% of participants were of White ethnicity. Although sampled from England and Wales alone, the sample represents the full socioeconomic spectrum of the UK population, as reflected in the families' distribution on neighbourhood‐level socioeconomic indices (Odgers, Caspi, Bates, Sampson, & Moffitt, 2012; Reuben et al., 2020) (see Appendix S1, Figure S1). Follow‐up home visits were conducted when the children were aged 7 (98% participation), 10 (96%), 12 (96%) and 18 years (93%). Visits at ages 5–12 included assessments with participants and their mother (primary caretaker) and at age 18 included interviews with participants. The Joint South London and Maudsley and the Institute of Psychiatry Research Ethics Committee approved each phase of the study. Parents gave informed consent, and participants gave assent between 5 and 12 years, then informed consent at age 18.

In this study, we sought to capitalise on the nested structure of the E‐Risk study within TEDS, as they have complementary strengths in the assessment of trauma‐related disorders and autism, respectively. The sample in these two studies became increasingly non‐overlapping over time because of the follow‐up methods used (in‐person assessment for the E‐Risk Study; telephone/online assessment for TEDS), which reduced the size of the sample with data available from both studies. We identified participants with complete data for PTSD from the E‐Risk study (*N* = 2,061) who had parent‐reported Childhood Autism Spectrum Test (CAST) (Scott, Baron‐Cohen, Bolton, & Brayne, 2002) from TEDS (*N* = 1,510). The majority had CAST data at age 8 (*n* = 1,213). Because CAST scores showed high correlations (*r* ~ .6; Appendix S2) and were stable (Appendix S2, Figure S2) between ages 8, 9 and 12 years, in order to maximise the analytical sample, we used available parent‐reported CAST scores at ages 9 or 12 where scores at age 8 were not available. This resulted in a sample of 1,510 E‐Risk Study members with complete data for both autistic traits and PTSD. Six participants (0.4% of the sample) had missing IQ data at age 5 and were removed from the analyses, resulting in the final analytical sample of 1,504 participants. This sample and the overall E‐Risk participants at age 18 showed similar distributions for variables included in the analyses (see Appendix S3, Table S1).

### Measures

#### Trauma exposure and PTSD measure

Trauma exposure and PTSD to date were assessed at age 18 years, during face‐to‐face interviews with the twin participants conducted by the E‐Risk Study team. Participants were asked whether they had been exposed to trauma during their lifetime, according to DSM‐5 PTSD Criterion A (American Psychiatric Association, 2013). Participants who reported trauma exposure were then asked to describe the trauma they had experienced. Participants who reported trauma exposure were also further evaluated through an adapted version of the Diagnostic Interview Schedule (Robins, Cottler, Bucholz, & Compton, 1995) to assess for current (past‐year) and lifetime PTSD according to the DSM‐5 criteria. Our analyses focus on E‐Risk Study members meeting criteria for lifetime PTSD, but results for current PTSD are also reported in sensitivity analyses detailed in the Appendix S6.

#### Autistic traits measure

The Childhood Autism Spectrum Test (CAST) (originally named ‘Childhood Asperger's Syndrome Test’) (Scott et al., 2002) was designed as a parent‐report screening instrument for autism in non‐clinical samples. The CAST is a quantitative scale; all questions are answered ‘yes’ or ‘no’ by the child's mother (or primary caregiver) and are scored additively. Where items had missing data, if participants' parents had completed more than half of the questions, the total score was calculated by summing the completed items and finding the average based on the total possible scores. CAST parent‐report total score at age 8 was selected as the primary measure of autistic traits, and to increase the coverage, missing scores were replaced with CAST scores taken when participants were 9 or 12 years old (see Appendix S2).

#### General psychopathology measure

The ‘*p*’ factor is a composite index of general psychopathology. The measure of ‘*p*’ was computed via a bi‐factor model that was fitted to 11 distinct symptom scales encompassing various mental health conditions (Schaefer et al., 2018). The symptoms were assessed in the E‐Risk Study during the age‐18 follow‐up with a past‐year reporting period including major depressive disorder, generalised anxiety disorder, PTSD, disordered eating, attention‐deficit hyperactivity disorder (ADHD), conduct disorder, alcohol dependence, cannabis dependence, nicotine dependence, psychotic symptoms and prodromal symptoms. A description of how each of these was assessed is provided in Appendix S4. After scaling the scores, the mean value was set to 100, and the standard deviation was fixed at 15.

#### Not in education, employment or training (NEET) status

At the time of their age‐18 interview, participants were classified as NEET if they reported that they were neither studying nor working in paid employment nor pursuing a vocational qualification or apprenticeship training. Participants were asked to ensure that NEET status was not simply a function of being on summer holiday or of being a parent. The employment‐ and education‐related questions were those used in the Longitudinal Study of Young People in England (LSYPE). This operationalisation of NEET status follows that used by the UK Office of National Statistics and the International Labour Organisation (Chandler & Barrett, 2013).

#### Potentially confounding variables

Covariates for the adjusted models were selected because previous literature reported a relationship with both autistic traits and PTSD, suggesting their potential role as confounders. In particular, previous research found that the prevalence of both autistic traits and PTSD varies according to sex (Lewis et al., 2019; Napolitano et al., 2022), IQ (Koenen, Moffitt, Poulton, Martin, & Caspi, 2007; Marinopoulou et al., 2025) and socio‐economic status (Lewis et al., 2019; Skylark & Baron‐Cohen, 2017). Biological sex and childhood IQ scores were obtained at first contact with the twins at age 5. IQ was assessed using Vocabulary and Block Design subtests on a short form of the Wechsler Preschool and Primary Scale of Intelligence‐Revised and prorated according to Sattler (1988). Family SES was defined through a standardised composite of parental income, education and occupation obtained from mothers at the age‐5 assessment (Trzesniewski, Moffitt, Caspi, Taylor, & Maughan, 2006). In the full E‐Risk dataset, the population‐wide distribution of this composite variable was then split into tertiles. In the present study, the variable was coded so that 1 indicates the highest socioeconomic status, and 3 indicates the lowest.

### Data analysis

CAST scores were log‐transformed to approximate a normal distribution. Bivariate logistic regression models were used to investigate the association of autistic traits with (1) report of trauma exposure (PTSD Criterion A), (2) PTSD diagnosis and (3) NEET status (from age 18 years assessment). Bivariate linear regression models were used to test the association of autistic traits with general psychopathology. These bivariate models were then expanded to account for the influence of possible confounding factors (SES, IQ and sex) in multivariate analyses, first accounting for each single potential confounder and then in a saturated model including all potential confounders at once. The analyses were first carried out in the overall sample and subsequently in the subsample of trauma‐exposed participants in order to test if any associations of autistic traits and outcomes could be explained by greater risk for trauma exposure in participants with higher autistic traits. Analysis code is available at the study's OSF pre‐registration (https://osf.io/uf9t7/).

We also undertook sensitivity analyses to test the possible biases introduced by our approach to missingness in CAST data and examining past‐year rather than lifetime PTSD diagnosis. Standard errors were adjusted to account for the non‐independence of twin observations (Williams, 2000) applying heteroskedasticity‐robust standard errors while clustering for membership in the same family. Because our analytical sample and the overall E‐Risk sample at age 18 showed similar distributions for variables included in the analyses (Appendix S3, Table S1), we carried out analyses on study members with complete data. None of the included variables breached multicollinearity thresholds (Appendix S5, Figure S4).

## Results

**Figure 1:**
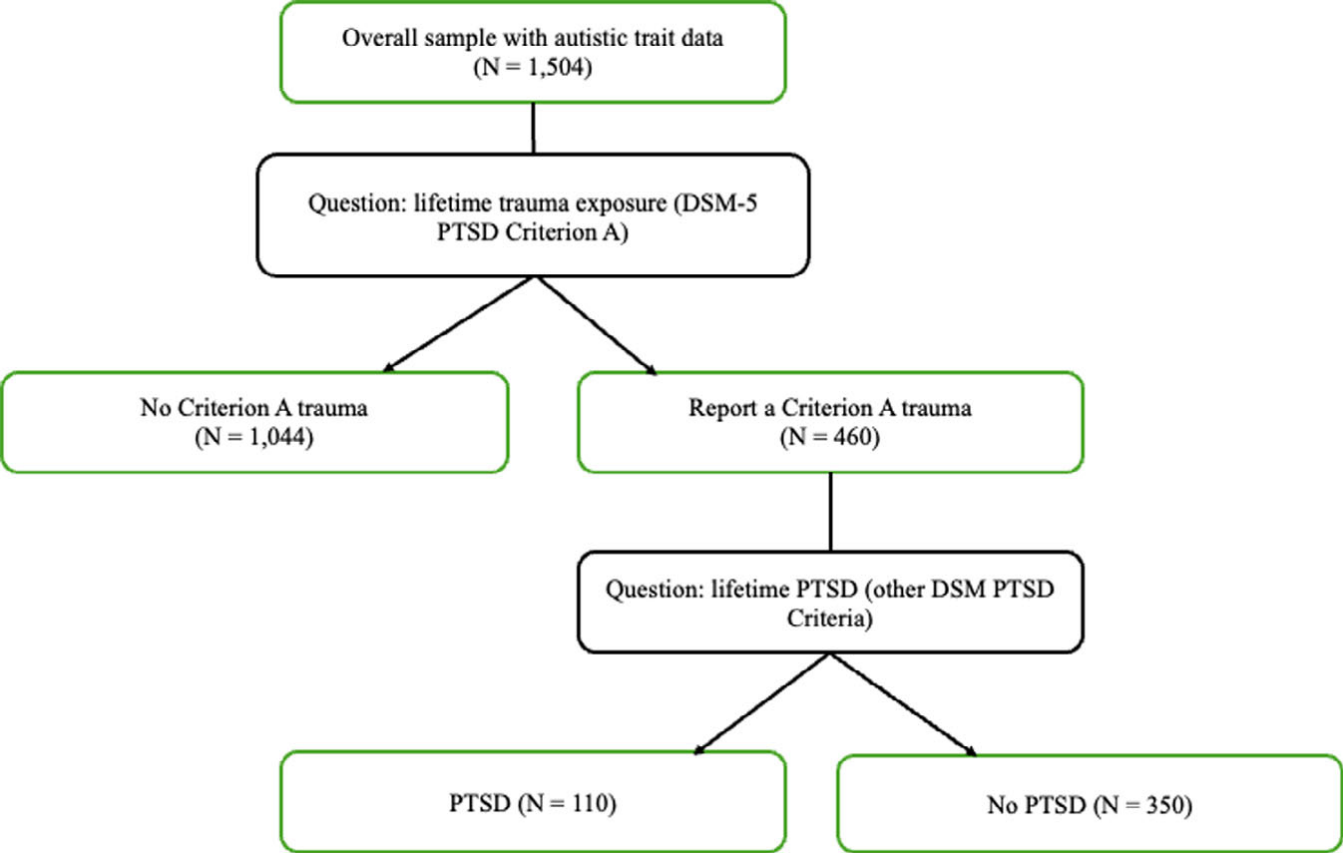
Flow chart illustrating sample selection

As shown in Figure 1, the overall sample consisted of 1,504 people, 460 of whom reported trauma exposure by age 18 and were therefore further assessed for PTSD. Of those who reported trauma exposure, 24% (*n* = 110) participants met PTSD diagnostic criteria. The overall analytical sample was 53.8% female (*n* = 809), had a mean CAST score of 5.47, a mean IQ of 102, and was similarly distributed in SES tertiles. Table 1 shows the demographic characteristics of the overall sample and trauma‐exposed subsample, as well as details on trauma‐unexposed participants, all participants with no PTSD, trauma‐exposed participants with no PTSD, and those with PTSD.

**Table 1:** Sample description

	Sample					
	Overall (*N* = 1,504)	Trauma exposed participants (*n* = 460)	Trauma unexposed participants (*n* = 1,044)	Overall sample with no PTSD (*n* = 1,394)	Trauma exposed with no PTSD (*n* = 350)	Trauma exposed with PTSD (*n* = 110)
Sex, *N* (%)						
Male	695 (46.2)	201 (43.7)	494 (47.3)	661 (47.4)	167 (47.7)	34 (30.9)
Female	809 (53.8)	259 (56.3)	550 (52.7)	733 (52.6)	183 (52.3)	76 (69.1)
SES, *N* (%)						
High SES	576 (38.3)	153 (33.3)	423 (40.5)	553 (39.7)	130 (37.1)	23 (20.9)
Medium SES	500 (33.2)	152 (33.0)	348 (33.3)	462 (33.1)	114 (32.6)	38 (34.5)
Low SES	428 (28.5)	155 (33.7)	273 (26.1)	379 (27.2)	106 (30.3)	49 (44.5)
NEET *N* (%)	151 (10.0)	71 (15.4)	80 (7.7)	125 (9.0)	45 (12.9)	84 (76.4)
Autistic traits (max = 31) mean (*SD*)	5.47 (3.5)	5.79 (3.6)	5.33 (3.5)	5.38 (3.5)	5.52 (3.5)	6.62 (3.9)
IQ mean (*SD*)	102 (14.9)	101 (14.2)	102 (15.2)	102 (14.9)	102 (14.1)	99.0 (14.4)
‘*p*’ factor mean (*SD*)	99.2 (14.7)	107.0 (15.8)	95.5 (12.5)	97.4 (13.3)	103.0 (13.9)	121.0 (13.7)

Data are *n*/*N* (%) unless stated otherwise. IQ, intelligence quotient; NEET, not in education, employment, or training; PTSD, post‐traumatic stress disorder; SES, socio‐economic status; ‘*p*’‐factor, measure of general psychopathology.

### Potential confounders

Associations of key potential confounders (i.e. sex, IQ and SES) with the predictor variable (autistic traits) and each outcome variable are reported in Appendix S5. In brief, children with autistic traits were less likely to be female in the overall sample. In both the overall and trauma‐exposed samples, autistic traits were associated with lower IQ. Associations between the potential confounders and outcome variables are shown in the first panels of Tables 2 and 3. In both the overall sample and the trauma‐exposed subsample, univariate analyses with potential confounders showed significant associations between low SES and all outcomes, and female sex with PTSD and general psychopathology.

**Table 2:** Results from the overall sample (*N* = 1,504); univariate models of autistic traits and confounding variables' individual relationships with outcomes at age 18, and multivariate models showing the association between autistic traits and outcomes of interest, adjusting for confounding variables

	Univariate models	Multivariate models adjusted for:			
		Sex	IQ	SES	Sex, IQ and SES
Panel A: Associations with trauma exposure in the overall sample‐OR [95% CI]					
Autistic traits	**1.26 [1.03–1.54]**	**1.29 [1.05–1.58]**	**1.23 [1.00–1.50]**	1.17 [0.96–1.44]	1.20 [0.97–1.48]
Female sex	1.16 [0.93–1.44]	*1.20 [0.96–1.50]*	–	–	*1.18 [0.94–1.48]*
IQ	0.99 [0.99–1.00]	–	*1.00 [0.99–1.00]*	–	*1.00 [0.99–1.01]*
Medium SES	1.21 [0.93–1.58]	–	–	*1.18 [0.90–1.54]*	*1.17 [0.89–1.55]*
Low SES	**1.57 [1.20–2.06]**	–	–	***1.49 [1.13–1.97]***	***1.46 [1.08–1.98]***
Panel B: Associations with PTSD diagnosis in the overall sample‐OR [95% CI]					
Autistic traits	**1.91 [1.29–2.82]**	**2.10 [1.40–3.14]**	**1.78 [1.20–2.64]**	**1.59 [1.08–2.33]**	**1.77 [1.17–2.66]**
Female sex	**2.02 [1.33–3.06]**	***2.24 [1.46–3.44]***	–	–	***2.21 [1.43–3.41]***
IQ	0.99 [0.97–1.00]	–	*0.99 [0.98–1.00]*	–	*1.00 [0.99–1.02]*
Medium SES	**1.98 [1.16–3.37]**	–	–	***1.85 [1.08–3.18]***	***1.89 [1.09–3.29]***
Low SES	**3.11 [1.86–5.19]**	–	–	***2.67 [1.58–4.53]***	***2.62 [1.49–4.59]***
Panel C: Associations with NEET status in the overall sample – OR [95% CI]					
Autistic traits	**1.48 [1.05–2.09]**	**1.53 [1.08–2.17]**	1.18 [0.85–1.64]	1.05 [0.76–1.47]	0.98 [0.70–1.37]
Female sex	1.22 [0.87–1.72]	*1.30 [0.92–1.84]*	–	–	*1.18 [0.82–1.70]*
IQ	0.96 [0.95–0.98]	–	*0.97 [0.95–0.98]*	–	*0.98 [0.96–0.99]*
Medium SES	1.51 [0.88–2.58]	–	–	*1.49 [0.87–2.58]*	*1.26 [0.73–2.19]*
Low SES	**6.20 [3.91–9.85]**	–	–	***6.10 [3.78–9.83]***	***4.68 [2.86–7.65]***
Panel D: Associations with the ‘*p*’‐factor in the overall sample – Beta [95% CI]					
Autistic traits	**3.22 [1.84–4.60]**	**3.53 [2.16–4.91]**	**2.79 [1.41–4.17]**	**2.46 [1.05–3.86]**	**2.65 [1.25–4.04]**
Female sex	**2.33 [0.85–3.80]**	***2.83 [1.36–4.29]***	*–*	*–*	***2.64 [1.17–4.11]***
IQ	**−0.10 [−0.15 to −0.05]**	–	***−0.08 [−0.13 to −0.03]***	*–*	*−0.04 [−0.09–0.02]*
Medium SES	**2.22 [0.53–3.91]**	*–*	*–*	***1.84 [0.13–3.55]***	*1.57 [−0.19–3.32]*
Low SES	**5.28 [3.42–7.14]**	*–*	*–*	***4.46 [2.55–6.37]***	***3.85 [1.79–5.90]***

Values in bold text indicate statistically significant results (*p* < .05). Values in Italic text are contributions of confounding variables to the association between autistic traits and outcomes of interest. All models are adjusted for the non‐independence of twin observations. 95% CI, 95% confidence intervals; Beta, beta coefficient; IQ, intelligence quotient; NEET, not in education, employment, or training; OR, odds ratio; ‘*p*’‐factor, measure of general psychopathology; PTSD, post‐traumatic stress disorder; SES, socio‐economic status.

**Table 3:** Results from the trauma‐exposed subsample (*n* = 460); univariate models of autistic traits and confounding variables' individual relationships with outcomes at age 18, and multivariate models showing associations between autistic traits and outcomes of interest adjusting for confounding variables

	Univariate models	Multivariate model adjusted for			
		Sex	IQ	SES	Sex, IQ and SES
Panel A: Associations with PTSD diagnosis in the trauma‐exposed subsample – OR [95% CI]					
Autistic traits	**1.75 [1.15–2.68]**	**1.86 [1.21–2.88]**	**1.66 [1.08–2.54]**	**1.52 [1.00–2.32]**	**1.62 [1.04–2.53]**
Female sex	**2.04 [1.29–3.22]**	***2.17 [1.36–3.46]***	*–*	–	***2.18 [1.35–3.53]***
IQ	0.99 [0.97–1.00]	–	*0.99 [0.98–1.01]*	–	*1.00 [0.98–1.02]*
Medium SES	**1.88 [1.06–3.36]**	–	–	***1.80 [1.00–3.24]***	***1.84 [1.00–3.37]***
Low SES	**2.61 [1.49–4.58]**	–	–	***2.29 [1.29–4.08]***	***2.29 [1.24–4.21]***
Panel B: Associations with NEET status in the trauma‐exposed subsample – OR [95% CI]					
Autistic traits	1.07 [0.65–1.77]	1.07 [0.64–1.77]	0.88 [0.56–1.39]	0.77 [0.46–1.29]	0.66 [0.40–1.08]
Female sex	0.94 [0.56–1.56]	*0.94 [0.56–1.58]*	–	–	*0.85 [0.49–1.47]*
IQ	0.96 [0.94–0.98]	–	*0.96 [0.94–0.98]*	–	*0.97 [0.95–0.99]*
Medium SES	1.19 [0.53–2.68]	–	–	*1.23 [0.54–2.80]*	*0.99 [0.42–2.35]*
Low SES	**4.81 [2.41–9.57]**	–	–	***5.26 [2.55–10.85]***	***4.14 [2.00–8.58]***
Panel C: Associations with the ‘*p*’‐factor in the trauma‐exposed sub sample – Beta [95% CI]					
Autistic traits	**3.36 [0.68–6.04]**	**3.58 [0.95–6.21]**	**2.68 [0.00–5.36]**	2.47 [−0.21–5.16]	2.35 [−0.28–4.99]
Female sex	**3.23 [0.30–6.16]**	***3.51 [0.60–6.41]***	–	–	***3.26 [0.34–6.18]***
IQ	**−0.16 [−0.26 ‐ ‐0.06]**	–	***−0.14 [−0.24 ‐ ‐0.03]***	–	*−0.10 [−0.21–0.01]*
Medium SES	2.43 [−1.06–5.92]	–	–	*2.14 [−1.38–5.66]*	*1.51 [−2.00–5.02]*
Low SES	**5.75 [2.27–9.24]**	–	–	***4.94 [1.36–8.52]***	***3.92 [0.13–7.70]***

Values in bold text indicate statistically significant results (*p* < .05). Values in Italic text are contributions of confounding variables to the association between autistic traits and outcomes of interest. All models are adjusted for the non‐independence of twin observations. 95% CI, 95% confidence intervals; Beta, beta coefficient; IQ, intelligence quotient; NEET, not in education, employment, or training; OR, odds ratio; ‘*p*’‐factor, measure of general psychopathology; PTSD, post‐traumatic stress disorder; SES, socio‐economic status.

### Associations of autistic traits with trauma exposure, PTSD, general psychopathology and functional impairment in the overall sample

**Figure 2:**
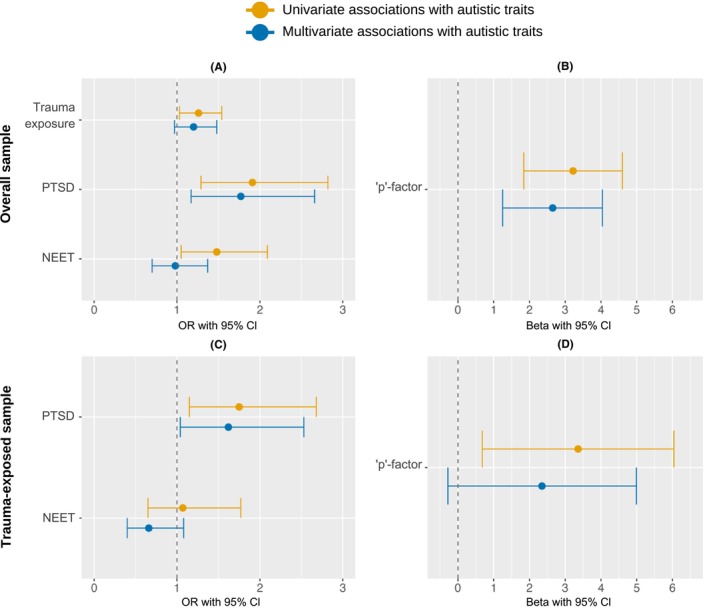
Univariate and multivariate regressions showing the association between autistic traits and outcome measures at age 18 for the overall sample (*N* = 1,504) and trauma‐exposed subsample (*n* = 460). All models are adjusted for the non‐independence of twin observations. Univariate regressions are from analyses unadjusted for potential confounders, and multivariate regression results are analyses accounting for confounding effects of sex, intelligence quotient and family socio‐economic status. Odds ratios and beta‐coefficients are plotted with 95% confidence limits. Figure 2 shows univariate and multivariate analyses between autistic traits and (A) trauma exposure, PTSD diagnosis and NEET status in the overall sample, (B) ‘*p*’‐factor in the overall sample, (C) PTSD diagnosis and NEET status in the trauma‐exposed subsample, and (D) ‘*p*’‐factor in the trauma‐exposed subsample. The dotted line on the axis indicates no association: 1 for logistic regressions in (A) and (C), and 0 for linear regressions (B) and (D). 95% CI, 95% confidence intervals; Beta, beta‐coefficient; NEET, not in education, employment or training; OR, odds ratio; ‘*p*’‐factor, measure of general psychopathology; PTSD, post‐traumatic stress disorder

Full results of analyses in the overall sample (*N* = 1,504) are presented in Table 2, and the main unadjusted and fully adjusted results are displayed in Figure 2A,B.

#### Trauma exposure

Children with higher autistic traits in childhood had a significantly increased likelihood of reporting exposure to traumatic events by 18 years (OR = 1.26, 95% CI = 1.03; 1.54; Table 2, Panel A). This association was attenuated and became statistically non‐significant when accounting for SES (OR = 1.17, 95% CI = 0.96; 1.44) and in the fully adjusted model (OR = 1.20, 95% CI = 0.97; 1.48).

#### PTSD diagnosis

Children with higher autistic traits had a significantly increased likelihood of meeting the diagnostic criteria for PTSD by the age of 18 (OR = 1.91, 95% CI = 1.29; 2.82; Table 2, Panel B). This association remained significant and was enhanced when sex (OR = 2.10, 95% CI = 1.40; 3.14) was accounted for, but was slightly attenuated when IQ (OR = 1.78, 95% CI = 1.20; 2.64) and SES (OR = 1.59, 95% CI = 1.08; 2.33) were accounted for individually and in the fully adjusted model (OR = 1.77, 95% CI = 1.17; 2.66).

#### General psychopathology

Children with higher autistic traits had a significantly increased likelihood of having more severe psychopathology at age 18 (Beta = 3.22, 95% CI = 1.84; 4.60; Table 2, Panel D). This statistically significant relationship was maintained when the analysis was individually adjusted for potential confounders; the association was enhanced when sex was accounted for (Beta = 3.53, 95% CI = 2.16; 4.91) and attenuated when adjusted for IQ (Beta = 2.79, 95% CI = 1.41; 4.17) or SES (Beta = 2.46, 95% CI = 1.05; 3.86). The association also remained significant, albeit slightly attenuated, in the fully adjusted analysis (Beta = 2.65, 95% CI = 1.25; 4.04).

#### Functional impairment

Children with higher autistic traits had a significantly increased likelihood of being NEET at age 18 (OR = 1.48, 95% CI = 1.05; 2.09; Table 2, Panel C). This association remained significant and slightly enhanced when accounting for sex (OR = 1.53, 95% CI = 1.08; 2.17). However, this association was attenuated and not statistically significant when IQ (OR = 1.18, 95% CI = 0.85; 1.64) and SES (OR = 1.05, 95% CI = 0.76; 1.47) were accounted for, nor when the model was adjusted for all possible confounders (OR = 0.98, 95% CI = 0.70; 1.37).

### Associations of autistic traits with PTSD, general psychopathology and functional impairment in the trauma‐exposed subsample

Full results of analyses in the trauma‐exposed subsample (*n* = 460) are given in Table 3 and the main unadjusted and fully adjusted results are displayed in Figure 2C,D.

#### PTSD diagnosis

In young people who reported trauma exposure, those with higher autistic traits had a significantly increased likelihood of meeting the PTSD diagnostic criteria by the age of 18 (OR = 1.75, 95% CI = 1.15; 2.68; Table 3, Panel A). This remained statistically significant when adjusted for confounders; the association was slightly enhanced when sex (OR = 1.86, 95% CI = 1.21; 2.88) was accounted for, and slightly attenuated when adjusting for IQ (OR = 1.66, 95% CI = 1.08; 2.54) and SES (OR = 1.52, 95% CI = 1.00; 2.32), as well as in the fully adjusted model (OR = 1.62, 95% CI = 1.04; 2.53).

#### General psychopathology

In young people who reported trauma exposure, those with higher autistic traits had a significantly increased likelihood of having higher levels of psychopathology at age 18 (Beta = 3.36, 95% CI = 0.68; 6.04; Table 3, Panel C). This relationship was not significant when the model was adjusted for confounders; the association was enhanced when sex was accounted for (Beta = 3.58, 95% CI = 0.95; 6.21), but attenuated when controlling for IQ (Beta = 2.68, 95% CI = 0.00; 5.36) or SES (Beta = 2.47, 95% CI = −0.21; 5.16), and in the fully adjusted model (Beta = 2.35, 95% CI = −0.28; 4.99).

#### Functional impairment

In young people who reported trauma exposure, those with higher autistic traits did not have a significantly increased likelihood of being NEET at age 18 (OR = 1.07, 95% CI = 0.65; 1.77; Table 3, Panel B). This remained statistically non‐significant when adjusting for confounders; accounting for sex had minimal impact on the association (OR = 1.07, 95% CI = 0.64; 1.77), whereas the association was attenuated to a (non‐significant) *decrease* in the likelihood of being NEET when adjusting for IQ (OR = 0.8, 95% CI = 0.56; 1.39) or SES (OR = 0.77, 95% CI = 0.46; 1.29), as well as in the fully adjusted model (OR = 0.66, 95% CI = 0.40; 1.08).

### Sensitivity analyses

First, we restricted the analytical sample to participants with complete CAST data at age 8 to test if the replacement of missing CAST data at age 8 impacted our findings. In the restricted sample with complete CAST data at age 8 (*n* = 1,213), we found similar results as in the overall sample used above (see Appendix S6, Tables S3, S4). Second, we focused the analyses predicting PTSD on the past‐year diagnosis, rather than the lifetime diagnosis used above, to provide a clearer timeline for the associations examined. As expected, fewer participants had a past‐year PTSD diagnosis (*n* = 63) than a lifetime diagnosis. Focusing on the past‐year PTSD diagnosis, we found similar results to those obtained in the analyses using the lifetime PTSD diagnosis presented above (see Appendix S6, Tables S5, S6).

## Discussion

Our findings in a longitudinal birth cohort indicate an elevated propensity for meeting diagnostic criteria for PTSD in young people with higher autistic traits in childhood, over and above the effects of established risk factors, such as female sex, lower IQ, and lower SES. The relationship between autistic traits and PTSD persisted in the subsample of young people who reported trauma exposure, suggesting that the association was not simply explained by an increased risk of trauma exposure but reflects greater vulnerability to develop PTSD in trauma‐exposed young people with higher autistic traits. Beyond PTSD, children with higher autistic traits in the overall sample had greater general psychopathology (‘*p*’), but this association was no longer significant in the subset of trauma‐exposed young people after accounting for potential confounding effects. Higher autistic traits in childhood also increased the likelihood of the young people reporting trauma exposure and being NEET at age 18, but these associations were no longer statistically significant after accounting for low SES.

Children with higher autistic traits had higher rates of self‐reported trauma exposure, but this association was statistically accounted for by co‐occurring disadvantaged socio‐economic conditions. This is in line with the strong association of socio‐economic disadvantage with childhood trauma and adversity (Lacey, Howe, Kelly‐Irving, Bartley, & Kelly, 2022). Because studies have consistently reported higher rates of trauma exposure among autistic versus non‐autistic adults (Quinton, Ali, Danese, Happé, & Rumball, 2024), we expected that autistic traits would be associated with reported trauma exposure in our study. However, the only case–control study that found higher rates of trauma exposure in autistic versus non‐autistic children did not take into account SES (Paul, Gallot, Lelouche, Bouvard, & Amestoy, 2018). Our findings suggest that socio‐economic factors may help explain the relationship between autistic traits and trauma exposure. It is also possible that the observed relationships between autistic traits, trauma exposure and socio‐economic disadvantage may be due to overlapping influences, and our observational design cannot conclusively determine a direct mechanistic role for socio‐economic disadvantage. Future research using experimental designs and/or repeated measures of socio‐economic status (Ludwig et al., 2011) might be able to disentangle the mechanisms underlying the association between autistic traits and trauma exposure. Furthermore, the current analyses tested whether autistic traits are associated with exposure to trauma, as narrowly defined in DSM‐5 (American Psychiatric Association, 2013). Research suggests that this narrow definition may not capture the broader set of negative experiences that young people with high autistic traits also appraise as traumatic and might trigger PTSD in this group (as seen in autistic adults, Rumball, Happé, & Grey, 2020). The role of subjective appraisal of trauma memories has been demonstrated as a key determinant of trauma‐related psychopathology (Baldwin, Coleman, Francis, & Danese, 2024; Coleman et al., 2024; Danese & Widom, 2020). More clinical and epidemiological research is needed to map and adapt definitions of trauma for neurodivergent young people, and these findings highlight the importance of an intersectional approach to investigating the association between autistic characteristics and trauma exposure.

Children with higher autistic traits were more likely to develop PTSD as young adults, regardless of the effects of sex, IQ and SES. Furthermore, the findings did not simply reflect a heightened risk of trauma exposure, as the association held within the trauma‐exposed subgroup. Rather, the findings suggest that trauma‐exposed children with higher autistic traits were more likely to develop PTSD than their low‐trait peers. As discussed in the introduction, studies largely undertaken in adult samples suggest an overlap between established cognitive vulnerabilities for PTSD and cognitive differences observed in autistic individuals. Further research is necessary to identify the specific profile of autistic characteristics that increase the likelihood of PTSD. By mapping cognitive risk factors linked with specific autistic traits in young people, we may better understand the mechanisms underlying the observed associations and provide more targeted support.

Autistic traits were associated with poorer mental health outcomes above and beyond PTSD, as measured by a general psychopathology factor, in line with other population‐based studies (Lundström et al., 2011). This was observed both in the overall sample and in the trauma‐exposed subsample to similar effect. However, after adjusting for potential confounders, the association remained significant only in the overall sample. The association in the overall sample is unsurprising given the wealth of research on poorer mental health outcomes for autistic people compared with non‐autistic people (Lai et al., 2019; Muniandy, Richdale, Arnold, Trollor, & Lawson, 2021), as well as people with high autistic traits compared with those with low/no autistic traits (Stewart et al., 2020). Lack of statistical significance in the adjusted association between autistic traits and general psychopathology within the trauma‐exposed subsample may reflect lower statistical power (the effect sizes in adjusted analyses were similar in the overall sample and trauma‐exposed subsample) or a stronger role of the potential confounders examined. Our research adds to previous findings by indicating that autistic traits might exacerbate the impact of childhood trauma on general psychopathology, although more research is needed to disentangle causal and non‐causal explanations.

Beyond mental health outcomes, our results showed a small association between autistic traits and NEET status at age 18 in the overall sample. However, this association became non‐significant when other risk factors were considered, suggesting that SES and IQ played a role in this relationship. No relationship was observed between autistic traits and NEET status in the trauma‐exposed subsample. There is minimal research on autistic traits and functional outcomes in the general population, but research with college students found autistic traits to be associated with academic difficulties, independent of an autism diagnosis (McLeod & Anderson, 2023). Furthermore, higher autistic traits have been associated with lower personal income in a sample of 2,491 adults (Skylark & Baron‐Cohen, 2017). We know that finding employment has additional challenges for autistic adults, including being burdened by the need to conceal or ‘camouflage’ their autistic traits (Finn, Flower, Leong, & Hedley, 2023). Autistic people are underrepresented in the labor market (Frank et al., 2018), and previous research has highlighted that poorer mental health is one reason that autistic people's employment (Chen, Leader, Sung, & Leahy, 2015) and education (Van Hees, Moyson, & Roeyers, 2015) are disrupted. Given the negative findings, it is possible that autistic traits in the general population do not have independent effects on broad functional outcomes, such as NEET, beyond the detrimental impact of low IQ, low SES and trauma exposure.

## Limitations

The first limitations pertain to the measure of childhood autistic traits. This measure was produced from CAST scores taken at different ages (mainly at 8 years, but also at 9 and 12 years when age‐8 scores were not available) to maximise the sample size, but CAST scores at later ages might not have provided a reliable proxy for earlier CAST scores. However, CAST scores at these ages showed moderate positive correlations in our sample, and previous work in the full TEDS cohort found that individual differences in autistic traits measured by parent‐reported CAST were stable from ages 8–12 years (Holmboe et al., 2014). Only a small number of children (*n* = 33, 2.19%) met the validated cut‐off (≥14) suggestive of potential autism (Williams et al., 2005), which is in line with a recent estimate that 1%–2% of the UK population are autistic (NHS Digital, 2020; Zeidan et al., 2022). While it is clear that high autistic traits do not equate to an autism diagnosis (Lord & Bishop, 2021), utilising a trait‐wise approach has proved beneficial for identifying and including those from under‐diagnosed groups, such as older adults (Stewart et al., 2023), women (Cardon, McQuarrie, Calton, & Gabrielsen, 2023) and those from low‐income countries (Heys et al., 2018). While useful, our findings may not generalise to autistic young people, and it will be essential to replicate our findings in a diagnosed autistic sample. Second, trauma exposure was measured by retrospective self‐report and was recorded without information on the specific timing of the exposure. It is possible that the onset of trauma‐related psychopathology preceded the autistic trait measure, and due to overlapping characteristics of autism and trauma responses in children (Stavropoulos, Bolourian, & Blacher, 2018), the exposure may have impacted the expression of the autistic trait score. However, sensitivity analysis focusing on PTSD diagnosis within the last 12 months (instead of lifetime) showed a very similar pattern of results. Third, there was a small overlap between the lifetime PTSD diagnosis and general psychopathology outcomes, as the *p*‐factor was calculated from the symptoms of 11 mental health conditions over the past year, including PTSD. Although the *p*‐factor and PTSD measures are not fully independent, we chose to use the previously published *p*‐factor measure (Schaefer et al., 2018) to ensure comparability with other E‐Risk studies and because PTSD likely makes a limited independent contribution to the *p*‐factor due to its relatively low prevalence and high rates of psychiatric comorbidity. Fourth, although we controlled in the analyses for key potential confounders (sex, IQ, SES), unmeasured confounders could provide alternative explanations for the associations observed. Finally, these data were from a sample of twins; it has been argued that the experiences of twins may not be representative of those of singletons, and findings may not be generalisable to people who are not twins (Røysamb & Tambs, 2016). However, the prevalence of trauma and psychiatric disorders in E‐Risk participants is within the range reported in studies involving non‐twins (Lewis et al., 2019; Lewis et al., 2021), supporting the wider applicability of findings from this cohort.

## Conclusions

This is the first longitudinal study to show a significant association between autistic traits in childhood and PTSD diagnosis by early adulthood. Future research needs to elucidate specific mechanisms of vulnerability to PTSD in young people with autistic traits, so that those at risk can be identified and receive targeted support in which their neurodivergent characteristics are accommodated.

## Ethical considerations

The Joint South London and Maudsley and the Institute of Psychiatry Research Ethics Committee approved each phase of the E‐Risk study. Parents gave written informed consent, and twins gave assent between 5 and 12 years; twins then gave informed consent at age 18. Ethical approval for TEDS was provided by the King's College London Ethics Committee (reference: PNM/09/10–104). Written informed consent was obtained from parents prior to data collection and from twins themselves from age 16 onward. The use of anonymised data for academic purposes did not require additional ethical approval.

**Key points**

- Cross‐sectional studies have previously shown positive associations between autistic traits and PTSD in adults.
- This is the first longitudinal study demonstrating a significant relationship between childhood autistic traits and meeting PTSD diagnostic criteria by early adulthood.
- Among the trauma‐exposed young people, autistic traits were also significantly associated with a lifetime PTSD diagnosis.
- Increased risk for trauma exposure amongst those with subclinical autistic traits is likely multi‐faceted and may be accounted for by factors such as socio‐economic status.
- Findings highlight the need for targeted assessments and evidence‐based treatments for PTSD in children with high autistic traits, and for future research to identify specific mechanisms of vulnerability to PTSD in autistic individuals.

## Supporting information


**Appendix S1.** Sample characteristics.
**Appendix S2.** Selecting the autistic trait measure.
**Appendix S3.** Missing data.
**Appendix S4.** Dimensional measures of psychopathology within the E‐Risk cohort at age 18.
**Appendix S5.** Associations between variables.
**Appendix S6.** Sensitivity analyses.

## Data Availability

Data for this study came from the Environmental Risk (E‐Risk) Longitudinal Twin Study and the Twins Early Development Study (TEDS). Researchers can apply for managed access to both (E‐Risk: https://eriskstudy.com/data‐access/; TEDS: https://www.teds.ac.uk/researchers/teds‐data‐access‐policy).

## References

[jcpp14163-bib-0001] American Psychiatric Association . (2013). Diagnostic and statistical manual of mental disorders (DSM‐5®) (5th edn). Author.

[jcpp14163-bib-0002] Baldwin, J.R. , Coleman, O. , Francis, E.R. , & Danese, A. (2024). Prospective and retrospective measures of child maltreatment and their association with psychopathology: A systematic review and meta‐analysis. JAMA Psychiatry, 81, 769–781. 38691376 10.1001/jamapsychiatry.2024.0818 PMC11063927

[jcpp14163-bib-0003] Best, C.S. , Moffat, V.J. , Power, M.J. , Owens, D.G.C. , & Johnstone, E.C. (2008). The boundaries of the cognitive phenotype of autism: Theory of mind, central coherence and ambiguous figure perception in young people with autistic traits. Journal of Autism and Developmental Disorders, 38, 840–847. 18004653 10.1007/s10803-007-0451-8

[jcpp14163-bib-0004] Brewin, C.R. , Dalgleish, T. , & Joseph, S. (1996). A dual representation theory of posttraumatic stress disorder. Psychological Review, 103, 670–686. 8888651 10.1037/0033-295x.103.4.670

[jcpp14163-bib-0005] Brosnan, M. , & Gavin, J. (2023). The impact of higher levels of autistic traits on risk of hikikomori (pathological social withdrawal) in young adults. PLoS One, 18, e0281833. 36809281 10.1371/journal.pone.0281833 PMC9942989

[jcpp14163-bib-0006] Cardon, G. , McQuarrie, M. , Calton, S. , & Gabrielsen, T.P. (2023). Similar overall expression, but different profiles, of autistic traits, sensory processing, and mental health between young adult males and females. Research in Autism Spectrum Disorders, 109, 102263. 37990737 10.1016/j.rasd.2023.102263 PMC10659573

[jcpp14163-bib-0007] Chandler, M. , & Barrett, R. (2013). UK estimate of young people not in education, employment or training. London: Office for National Statistics.

[jcpp14163-bib-0008] Chen, J.L. , Leader, G. , Sung, C. , & Leahy, M. (2015). Trends in employment for individuals with Autism Spectrum Disorder: A review of the research literature. Review Journal of Autism and Developmental Disorders, 2, 115–127.

[jcpp14163-bib-0009] Coleman, O. , Baldwin, J.R. , Dalgleish, T. , Rose‐Clarke, K. , Widom, C.S. , & Danese, A. (2024). Research Review: Why do prospective and retrospective measures of maltreatment differ? A narrative review. Journal of Child Psychology and Psychiatry, 65, 1662–1677. 39150090 10.1111/jcpp.14048 PMC11834142

[jcpp14163-bib-0070] Dahoun, T. , Peel, A. , Baldwin, J. , Coleman, O. , Lewis, S.J. , Wertz, J. , … & Danese, A. (2024). Genetic and environment influences on childhood victimization: A systematic review and meta‐analysis. Molecular Psychiatry. 10.1038/s41380-024-02868-z PMC12014478 39663379

[jcpp14163-bib-0010] Danese, A. , McLaughlin, K.A. , Samara, M. , & Stover, C.S. (2020). Psychopathology in children exposed to trauma: Detection and intervention needed to reduce downstream burden. British Medical Journal, 371, m3073. 33214140 10.1136/bmj.m3073 PMC7673907

[jcpp14163-bib-0011] Danese, A. , & Widom, C.S. (2020). Objective and subjective experiences of child maltreatment and their relationships with psychopathology. Nature Human Behaviour, 4, 811–818. 10.1038/s41562-020-0880-3 32424258

[jcpp14163-bib-0012] Ehlers, A. , & Clark, D.M. (2000). A cognitive model of posttraumatic stress disorder. Behaviour Research and Therapy, 38, 319–345. 10761279 10.1016/s0005-7967(99)00123-0

[jcpp14163-bib-0013] Finn, M. , Flower, R.L. , Leong, H.M. , & Hedley, D. (2023). ‘If I'm just me, I doubt I'd get the job’: A qualitative exploration of autistic people's experiences in job interviews. Autism, 27, 2086–2097. 36794473 10.1177/13623613231153480

[jcpp14163-bib-0014] Frank, F. , Jablotschkin, M. , Arthen, T. , Riedel, A. , Fangmeier, T. , Hölzel, L.P. , & van Tebartz Elst, L. (2018). Education and employment status of adults with autism spectrum disorders in Germany – A cross‐sectional‐survey. BMC Psychiatry, 18, 1–10. 29580218 10.1186/s12888-018-1645-7 PMC5870494

[jcpp14163-bib-0015] Golan, O. , Haruvi‐Lamdan, N. , Laor, N. , & Horesh, D. (2022). The comorbidity between autism spectrum disorder and post‐traumatic stress disorder is mediated by brooding rumination. Autism, 26, 538–544. 34318687 10.1177/13623613211035240

[jcpp14163-bib-0016] Happé, F. , & Frith, U. (2006). The weak coherence account: Detail‐focused cognitive style in autism spectrum disorders. Journal of Autism and Developmental Disorders, 36, 5–25. 16450045 10.1007/s10803-005-0039-0

[jcpp14163-bib-0069] Happé, F. , & Frith, U. (2020). Annual Research Review: Looking back to look forward – changes in the concept of autism and implications for future research. Journal of Child Psychology and Psychiatry, 61, 218–232. 10.1111/jcpp.13176 31994188

[jcpp14163-bib-0018] Haruvi‐Lamdan, N. , Lebendiger, S. , Golan, O. , & Horesh, D. (2019). Are PTSD and autistic traits related? An examination among typically developing Israeli adults. Comprehensive Psychiatry, 89, 22–27. 30579126 10.1016/j.comppsych.2018.11.004

[jcpp14163-bib-0019] Heys, M. , Gibbons, F. , Haworth, E. , Medeiros, E. , Tumbahangphe, K.M. , Wickenden, M. , … & Pellicano, E. (2018). The estimated prevalence of autism in school‐aged children living in rural Nepal using a population‐based screening tool. Journal of Autism and Developmental Disorders, 48, 3483–3498. 29855757 10.1007/s10803-018-3610-1 PMC6153945

[jcpp14163-bib-0020] Holmboe, K. , Rijsdijk, F.V. , Hallett, V. , Happé, F. , Plomin, R. , & Ronald, A. (2014). Strong genetic influences on the stability of autistic traits in childhood. Journal of the American Academy of Child and Adolescent Psychiatry, 53, 221–230. 24472256 10.1016/j.jaac.2013.11.001 PMC3919213

[jcpp14163-bib-0021] Hoover, D.W. , & Kaufman, J. (2018). Adverse childhood experiences in children with autism spectrum disorder. Current Opinion in Psychiatry, 31, 128–132. 29206686 10.1097/YCO.0000000000000390 PMC6082373

[jcpp14163-bib-0022] Huckins, L.M. , Johnson, J.S. , Cancelmo, L. , Diab, O. , Schaffer, J. , Cahn, L. , … & Feder, A. (2021). Polygenic regulation of PTSD severity and outcomes among World Trade Center responders. *medRxiv*. 10.1101/2020.12.06.20244772

[jcpp14163-bib-0024] Kerns, C.M. , Newschaffer, C.J. , & Berkowitz, S.J. (2015). Traumatic childhood events and autism Spectrum disorder. Journal of Autism and Developmental Disorders, 45, 3475–3486. 25711547 10.1007/s10803-015-2392-y

[jcpp14163-bib-0025] Kerns, C.M. , Rast, J.E. , & Shattuck, P.T. (2020). Prevalence and correlates of caregiver‐reported mental health conditions in youth with autism spectrum disorder in the United States. Journal of Clinical Psychiatry, 82, 11637. 10.4088/JCP.20m13242 33356021

[jcpp14163-bib-0026] Koenen, K.C. , Moffitt, T.E. , Poulton, R. , Martin, J. , & Caspi, A. (2007). Early childhood factors associated with the development of post‐traumatic stress disorder: Results from a longitudinal birth cohort. Psychological Medicine, 37, 181–192. 17052377 10.1017/S0033291706009019 PMC2254221

[jcpp14163-bib-0027] Lacey, R.E. , Howe, L.D. , Kelly‐Irving, M. , Bartley, M. , & Kelly, Y. (2022). The clustering of adverse childhood experiences in the Avon Longitudinal Study of Parents and Children: Are gender and poverty important? Journal of Interpersonal Violence, 37, 2218–2241. 32639853 10.1177/0886260520935096 PMC8918866

[jcpp14163-bib-0028] Lai, M.‐C. , Kassee, C. , Besney, R. , Bonato, S. , Hull, L. , Mandy, W. , … & Ameis, S.H. (2019). Prevalence of co‐occurring mental health diagnoses in the autism population: A systematic review and meta‐analysis. The Lancet Psychiatry, 6, 819–829. 31447415 10.1016/S2215-0366(19)30289-5

[jcpp14163-bib-0030] Lewis, S.J. , Arseneault, L. , Caspi, A. , Fisher, H.L. , Matthews, T. , Moffitt, T.E. , … & Danese, A. (2019). The epidemiology of trauma and post‐traumatic stress disorder in a representative cohort of young people in England and Wales. Lancet Psychiatry, 6, 247–256. 30798897 10.1016/S2215-0366(19)30031-8 PMC6384243

[jcpp14163-bib-0031] Lewis, S.J. , Koenen, K.C. , Ambler, A. , Arseneault, L. , Caspi, A. , Fisher, H.L. , … & Danese, A. (2021). Unravelling the contribution of complex trauma to psychopathology and cognitive deficits: A cohort study. The British Journal of Psychiatry, 219, 448–455. 34538875 10.1192/bjp.2021.57 PMC7611677

[jcpp14163-bib-0032] Lord, C. , & Bishop, S.L. (2021). Let's be clear that “Autism spectrum disorder symptoms” are not always related to autism spectrum disorder. American Journal of Psychiatry, 178, 680–682. 34383567 10.1176/appi.ajp.2021.21060578

[jcpp14163-bib-0073] Ludwig, J. , Sanbonmatsu, L. , Gennetian, L. , Adam, E. , Duncan, G.J. , Katz, L.F. , … & McDade, T.W. (2011). Neighborhoods, obesity, and diabetes – A randomized social experiment. The New England Journal of Medicine, 365, 1509–1519. 22010917 10.1056/NEJMsa1103216 PMC3410541

[jcpp14163-bib-0033] Lundström, S. , Chang, Z. , Kerekes, N. , Gumpert, C.H. , Råstam, M. , Gillberg, C. , … & Anckarsäter, H. (2011). Autistic‐like traits and their association with mental health problems in two nationwide twin cohorts of children and adults. Psychological Medicine, 41, 2423–2433. 21426604 10.1017/S0033291711000377

[jcpp14163-bib-0072] Marinopoulou, M. , Billstedt, E. , Wessman, C. , Bornehag, C.G. , & Hallerbäck, M.U. (2025). Association between intellectual functioning and autistic traits in the general population of children. Child Psychiatry and Human Development, 56, 264–275. 10.1007/s10578-023-01562-5 37351708 PMC11828797

[jcpp14163-bib-0034] Mazefsky, C.A. , Borue, X. , Day, T.N. , & Minshew, N.J. (2014). Emotion regulation patterns in adolescents with high‐functioning autism spectrum disorder: Comparison to typically developing adolescents and association with psychiatric symptoms. Autism Research, 7, 344–354. 24610869 10.1002/aur.1366 PMC4136477

[jcpp14163-bib-0035] McLeod, J.D. , & Anderson, E.M. (2023). Autistic traits and college adjustment. Journal of Autism and Developmental Disorders, 53, 3475–3492. 35796912 10.1007/s10803-022-05632-w PMC10465676

[jcpp14163-bib-0036] Moffitt, T.E. , & E‐Risk Study Team . (2002). Teen‐aged mothers in contemporary Britain. Journal of Child Psychology and Psychiatry, 43, 727–742. 12236608 10.1111/1469-7610.00082

[jcpp14163-bib-0037] Muniandy, M. , Richdale, A.L. , Arnold, S.R.C. , Trollor, J.N. , & Lawson, L.P. (2021). Inter‐relationships between trait resilience, coping strategies, and mental health outcomes in autistic adults. Autism Research, 14, 2156–2168. 34184818 10.1002/aur.2564

[jcpp14163-bib-0038] Napolitano, A. , Schiavi, S. , La Rosa, P. , Rossi‐Espagnet, M.C. , Petrillo, S. , Bottino, F. , … & Vicari, S. (2022). Sex differences in autism spectrum disorder: Diagnostic, neurobiological, and behavioral features. Frontiers in Psychiatry, 13, 889636. 35633791 10.3389/fpsyt.2022.889636 PMC9136002

[jcpp14163-bib-0039] NHS Digital . (2020). Autism waiting time statistics. NDRS. Available from: https://digital.nhs.uk/data‐and‐information/publications/statistical/autism‐statistics/q1‐april‐to‐june‐2020‐21/data‐quality‐copy

[jcpp14163-bib-0040] Odgers, C.L. , Caspi, A. , Bates, C.J. , Sampson, R.J. , & Moffitt, T.E. (2012). Systematic social observation of children's neighborhoods using Google Street View: A reliable and cost‐effective method. Journal of Child Psychology and Psychiatry, 53, 1009–1017. 22676812 10.1111/j.1469-7610.2012.02565.x PMC3537178

[jcpp14163-bib-0041] Paul, A. , Gallot, C. , Lelouche, C. , Bouvard, M.P. , & Amestoy, A. (2018). Victimisation in a French population of children and youths with autism spectrum disorder: A case control study. Child and Adolescent Psychiatry and Mental Health, 12, 48. 30524501 10.1186/s13034-018-0256-x PMC6276214

[jcpp14163-bib-0042] Peel, A.J. , Purves, K.L. , Baldwin, J.R. , Breen, G. , Coleman, J.R.I. , Pingault, J.B. , … & Eley, T.C. (2022). Genetic and early environmental predictors of adulthood self‐reports of trauma. British Journal of Psychiatry, 221, 613–620. 10.1192/bjp.2021.207 35105391

[jcpp14163-bib-0043] Quinton, A.M.G. , Ali, D. , Danese, A. , Happé, F. , & Rumball, F. (2024). The assessment and treatment of post‐traumatic stress disorder in autistic people: A systematic review. Review Journal of Autism and Developmental Disorders. 10.1007/s40489-024-00430-9 PMC13002758 41868251

[jcpp14163-bib-0044] Ratanatharathorn, A. , Koenen, K.C. , Chibnik, L.B. , Weisskopf, M.G. , Rich‐Edwards, J.W. , & Roberts, A.L. (2021). Polygenic risk for autism, attention‐deficit hyperactivity disorder, schizophrenia, major depressive disorder, and neuroticism is associated with the experience of childhood abuse. Molecular Psychiatry, 26, 1696–1705. 33483690 10.1038/s41380-020-00996-w PMC8164961

[jcpp14163-bib-0045] Reuben, A. , Sugden, K. , Arseneault, L. , Corcoran, D.L. , Danese, A. , Fisher, H.L. , … & Caspi, A. (2020). Association of neighborhood disadvantage in childhood with DNA methylation in young adulthood. The Journal of the American Medical Association (JAMA) Network Open, 3, e206095. 10.1001/jamanetworkopen.2020.6095 PMC7265095 32478847

[jcpp14163-bib-0046] Robins, L. , Cottler, L. , Bucholz, K. , & Compton, W. (1995). Diagnostic Interview Schedule for DSM‐IV. St. Louis: Washington University School of Medicine.

[jcpp14163-bib-0047] Robinson, E.B. , Koenen, K.C. , McCormick, M.C. , Munir, K. , Hallett, V. , Happé, F. , … & Ronald, A. (2011). Evidence that autistic traits show the same etiology in the general population and at the quantitative extremes (5%, 2.5%, and 1%). Archives of General Psychiatry, 68, 1113–1121. 22065527 10.1001/archgenpsychiatry.2011.119 PMC3708488

[jcpp14163-bib-0048] Røysamb, E. , & Tambs, K. (2016). The beauty, logic and limitations of twin studies. Norsk Epidemiologi, 26, 35–46.

[jcpp14163-bib-0071] Rumball, F. , Antal, K. , Happé, F. , & Grey, N. (2021). Co‐occurring mental health symptoms and cognitive processes in trauma‐exposed ASD adults. Research in Developmental Disabilities, 110, 103836. doi: 10.1016/j.ridd.2020.103836. 33453693 10.1016/j.ridd.2020.103836

[jcpp14163-bib-0049] Rumball, F. , Brook, L. , Happé, F. , & Karl, A. (2021). Heightened risk of posttraumatic stress disorder in adults with autism spectrum disorder: The role of cumulative trauma and memory deficits. Research in Developmental Disabilities, 110, 103848. 33454451 10.1016/j.ridd.2020.103848

[jcpp14163-bib-0050] Rumball, F. , Happé, F. , & Grey, N. (2020). Experience of trauma and PTSD symptoms in autistic adults: Risk of PTSD development following DSM‐5 and non‐DSM‐5 traumatic life events. Autism Research, 13, 2122–2132. 32319731 10.1002/aur.2306

[jcpp14163-bib-0051] Sallis, H.M. , Croft, J. , Havdahl, A. , Jones, H.J. , Dunn, E.C. , Smith, G.D. , … & Munafò, M.R. (2021). Genetic liability to schizophrenia is associated with exposure to traumatic events in childhood. Psychological Medicine, 51, 1814–1821. 32234096 10.1017/S0033291720000537 PMC8381289

[jcpp14163-bib-0052] Sattler, J.M. (1988). In 3rd edn (Ed.), Assessment of children. Jerome M. Sattler.

[jcpp14163-bib-0053] Schaefer, J.D. , Moffitt, T.E. , Arseneault, L. , Danese, A. , Fisher, H.L. , Houts, R. , … & Caspi, A. (2018). Adolescent victimization and early‐adult psychopathology: Approaching causal inference using a longitudinal twin study to rule out noncausal explanations. Clinical Psychological Science, 6, 352–371. 29805917 10.1177/2167702617741381 PMC5952301

[jcpp14163-bib-0054] Scott, F.J. , Baron‐Cohen, S. , Bolton, P. , & Brayne, C. (2002). The CAST (Childhood asperger syndrome test): Preliminary development of a UK screen for mainstream primary‐school‐age children. Autism, 6, 9–31. 11918111 10.1177/1362361302006001003

[jcpp14163-bib-0055] Skylark, W.J. , & Baron‐Cohen, S. (2017). Initial evidence that non‐clinical autistic traits are associated with lower income. Molecular Autism, 8, 61. 29158888 10.1186/s13229-017-0179-z PMC5683395

[jcpp14163-bib-0057] Stavropoulos, K.K.‐M. , Bolourian, Y. , & Blacher, J. (2018). Differential diagnosis of autism spectrum disorder and post traumatic stress disorder: Two clinical cases. Journal of Clinical Medicine, 7, 71. 29642485 10.3390/jcm7040071 PMC5920445

[jcpp14163-bib-0058] Stewart, G.R. , Corbett, A. , Ballard, C. , Creese, B. , Aarsland, D. , Hampshire, A. , … & Happé, F. (2023). The cognitive profile of middle‐aged and older adults with high vs. low autistic traits. Autism Research, 16, 429–440. 36454212 10.1002/aur.2866 PMC10947177

[jcpp14163-bib-0059] Stewart, G.R. , Corbett, A. , Ballard, C. , Creese, B. , Aarsland, D. , Hampshire, A. , … & Happé, F. (2020). Sleep problems and mental health difficulties in older adults who endorse high autistic traits. Research in Autism Spectrum Disorders, 77, 101633.

[jcpp14163-bib-0060] Trouton, A. , Spinath, F.M. , & Plomin, R. (2002). Twins early development study (TEDS): A multivariate, longitudinal genetic investigation of language, cognition and behavior problems in childhood. Twin Research, 5, 444–448. 12537874 10.1375/136905202320906255

[jcpp14163-bib-0061] Trzesniewski, K.H. , Moffitt, T.E. , Caspi, A. , Taylor, A. , & Maughan, B. (2006). Revisiting the association between reading achievement and antisocial behavior: New evidence of an environmental explanation from a twin study. Child Development, 77, 72–88. 16460526 10.1111/j.1467-8624.2006.00857.x

[jcpp14163-bib-0062] Van Hees, V. , Moyson, T. , & Roeyers, H. (2015). Higher education experiences of students with autism spectrum disorder: Challenges, benefits and support needs. Journal of Autism and Developmental Disorders, 45, 1673–1688. 25448918 10.1007/s10803-014-2324-2

[jcpp14163-bib-0063] Wang, Y. , Zhang, Y. , Liu, L. , Cui, J. , Wang, J. , Shum, D.H.K. , … & Chan, R.C.K. (2017). A meta‐analysis of working memory impairments in autism spectrum disorders. Neuropsychology Review, 27, 46–61. 28102493 10.1007/s11065-016-9336-y

[jcpp14163-bib-0064] Warrier, V. , & Baron‐Cohen, S. (2021). Childhood trauma, life‐time self‐harm, and suicidal behaviour and ideation are associated with polygenic scores for autism. Molecular Psychiatry, 26, 1670–1684. 31659270 10.1038/s41380-019-0550-x PMC8159746

[jcpp14163-bib-0065] Weiland, R.F. , Polderman, T.J. , Hoekstra, R.A. , Smit, D.J. , & Begeer, S. (2020). The Dutch Sensory Perception Quotient‐Short in adults with and without autism. Autism, 24, 2071–2080. 32720830 10.1177/1362361320942085 PMC7543020

[jcpp14163-bib-0066] Williams, J. , Scott, F. , Stott, C. , Allison, C. , Bolton, P. , Baron‐Cohen, S. , & Brayne, C. (2005). The CAST (Childhood Asperger Syndrome Test): Test accuracy. Autism, 9, 45–68. 15618262 10.1177/1362361305049029

[jcpp14163-bib-0067] Williams, R.L. (2000). A note on robust variance estimation for cluster‐correlated data. Biometrics, 56, 645–646. 10877330 10.1111/j.0006-341x.2000.00645.x

[jcpp14163-bib-0068] Zeidan, J. , Fombonne, E. , Scorah, J. , Ibrahim, A. , Durkin, M.S. , Saxena, S. , … & Elsabbagh, M. (2022). Global prevalence of autism: A systematic review update. Autism Research, 15, 778–790. 35238171 10.1002/aur.2696 PMC9310578

