# Supplementary material for: Autistic traits in childhood and post‐traumatic stress disorder as young adults: a cohort study
Source: J Child Psychol Psychiatry. 2025 Mar 25;66(10):1514–25. doi: 10.1111/jcpp.14163 (PMC12353592; doi:10.1111/jcpp.14163)
Supplement: Supplementary file 1 — Appendix S1. Sample characteristics. Appendix S2. Selecting the autistic trait measure. Appendix S3. Missing data. Appendix S4. Dimensional measures of psychopathology within the E‐Risk cohort at age 18. Appendix S5. Associations between variables. Appendix S6. Sensitivity analyses. [file JCPP-66-1514-s001.docx]

# Supporting information

**Quinton *et al.* Autistic traits in childhood and Post-Traumatic Stress Disorder as Young Adults: A Cohort Study.**

[Appendix S1: Sample Characteristics 2](#_Toc190259155)

[E-Risk 2](#_Toc190259156)

[Figure S1 4](#_Toc190259157)

[Appendix S2: Selecting the Autistic trait Measure 5](#_Toc190259158)

[Childhood Autism Spectrum Test (CAST) 5](#_Toc190259159)

[Figure S2 6](#_Toc190259160)

[Figure S3 7](#_Toc190259161)

[Table S1 8](#_Toc190259162)

[Appendix S3: Missing data 9](#_Toc190259163)

[Missing autistic trait data 9](#_Toc190259164)

[Table S2 9](#_Toc190259165)

[Appendix S4: Dimensional measures of psychopathology within the E-Risk cohort at age 18 10](#_Toc190259166)

[Assessment of symptoms of mental health conditions 10](#_Toc190259167)

[The structure of psychopathology 11](#_Toc190259168)

[Figure S3 13](#_Toc190259169)

[Appendix S5: Associations between variables 15](#_Toc190259170)

[Multicollinearity in predictor, confounding and outcome variables 15](#_Toc190259171)

[Figure S4 15](#_Toc190259172)

[Associations between potential confounders and autistic traits 16](#_Toc190259173)

[Appendix S6: Sensitivity analyses 17](#_Toc190259174)

[Sensitivity analysis: Using CAST score data at age 8 only 17](#_Toc190259175)

[Table S3 18](#_Toc190259176)

[Table S4 20](#_Toc190259177)

[Sensitivity analysis: Using PTSD within 12 months of assessment as outcome measure 22](#_Toc190259178)

[Table S5 23](#_Toc190259179)

[Table S6 24](#_Toc190259180)

[References 25](#_Toc190259181)

## Appendix S1: Sample Characteristics

### E-Risk

Participants were members of the Environmental Risk (E-Risk) Longitudinal Twin Study, which tracks the development of a birth cohort of 2232 British children. The sample was drawn from a larger birth register of twins born in England and Wales in 1994–1995 (Trouton et al., 2002). Full details about the sample are reported elsewhere (Moffitt & E-Risk Study Team, 2002). Briefly, the E-Risk sample was constructed in 1999–2000, when 1116 families (93% of those eligible) with same-sex 5-year-old twins participated in home visit assessments. This sample comprised 56% monozygotic (MZ) and 44% dizygotic (DZ) twin pairs; sex was evenly distributed within zygosity (49% male). Families were recruited to represent the UK population of families with new-borns in the 1990s, on the basis of residential location throughout England and Wales and mother’s age. Teenaged mothers with twins were over selected to replace high-risk families who were selectively lost to the register through non-response. Older mothers having twins via assisted reproduction were under selected to avoid an excess of well-educated older mothers. The study sample represents the full range of socioeconomic conditions in the UK, as reflected in the families’ distribution on a neighbourhood-level socioeconomic index (called ACORN (A Classification of Residential Neighbourhoods), developed by CACI Inc. for commercial use in Great Britain) (Odgers et al., 2012): 25.6% of E-Risk families live in ‘wealthy achiever’ neighbourhoods compared with 25.3% nationwide; 5.3% live in ‘urban prosperity’ neighbourhoods compared with 11.6% nationwide; 29.6% live in ‘comfortably off’ neighbourhoods compared with 26.9% nationwide; 13.4% live in ‘moderate means’ neighbourhoods compared with 13.9% nationwide; and 26.1% live in ‘hard-pressed’ neighbourhoods compared with 20.7% nationwide. E-Risk underrepresents ‘urban prosperity’ neighbourhoods because such households are likely to be childless.

Follow-up home visits were conducted when the children were aged 7 (98% participation), 10 (96% participation), 12 (96% participation) and 18 (93% participation) years. Home visits at ages 5, 7, 10 and 12 years included assessments with participants and their mother (or primary care-taker); the home visit at age 18 years included interviews only with participants. Each twin participant was assessed by a different interviewer. There were 2066 children who participated in the E-Risk assessments at age 18 years, and the proportions of MZ (55%) and male same-sex (47%) twins were almost identical to those found in the original sample at age 5 years. The average age of the twins at the time of assessment was 18.4 years (s.d. 0.36); all interviews were conducted after their 18th birthday. The study sample at age 18 years was equally distributed across all deciles of the Index of Multiple Deprivation 2015, which measures relative levels of deprivation in small areas in England (Figure S1).

### ****Figure S1****

***Population representativeness of the E-Risk Study***


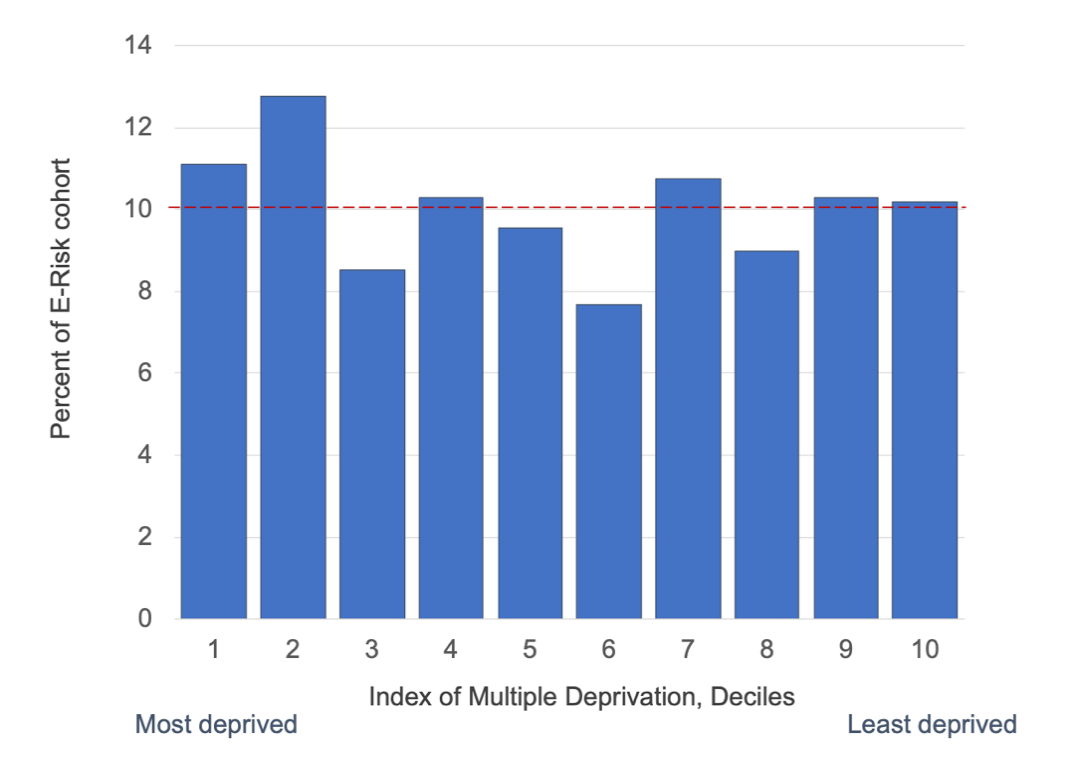


The histogram shows that E-Risk families’ addresses at age 18 years are a near-perfect match to the deciles of England’s Lower-layer Super Output Area (LSOA) Index of Multiple Deprivation 2015 (IMD) which averages 1,500 residents; approximately 10% of the cohort fills each of IMD’s 10% bands for England.

## Appendix S2: Selecting the Autistic trait Measure

### ****Childhood Autism Spectrum Test (****CAST)

**Childhood Autism Spectrum Test (**CAST) scores collected from parents when the child was age 8, 9 and 12 showed high correlations (Figure S2), and the mean scores were stable across the different ages (Figure S2). We removed participants who did not have parent reported CAST data for any of the three time points and examined the missingness of CAST data at ages 8, 9 and 12 in this sample (N=1504). Table S1 shows that CAST at age 8 was the most informative and was therefore selected as the primary autistic trait measure. In this sample, 1213 participants had data at age 8. For those with missing data at age 8, a replacement method was used to maximise the analytical sample size. Where possible, missing data was replaced with CAST score at age 12 (n= 242), as this was the most highly correlated with age 8 (r=0.6). The remaining participants with missing data (n=49) were replaced with CAST scores at age 9.

### ****Figure S2****

***Correlations between Childhood Autism Spectrum Test (CAST) scores at different ages***


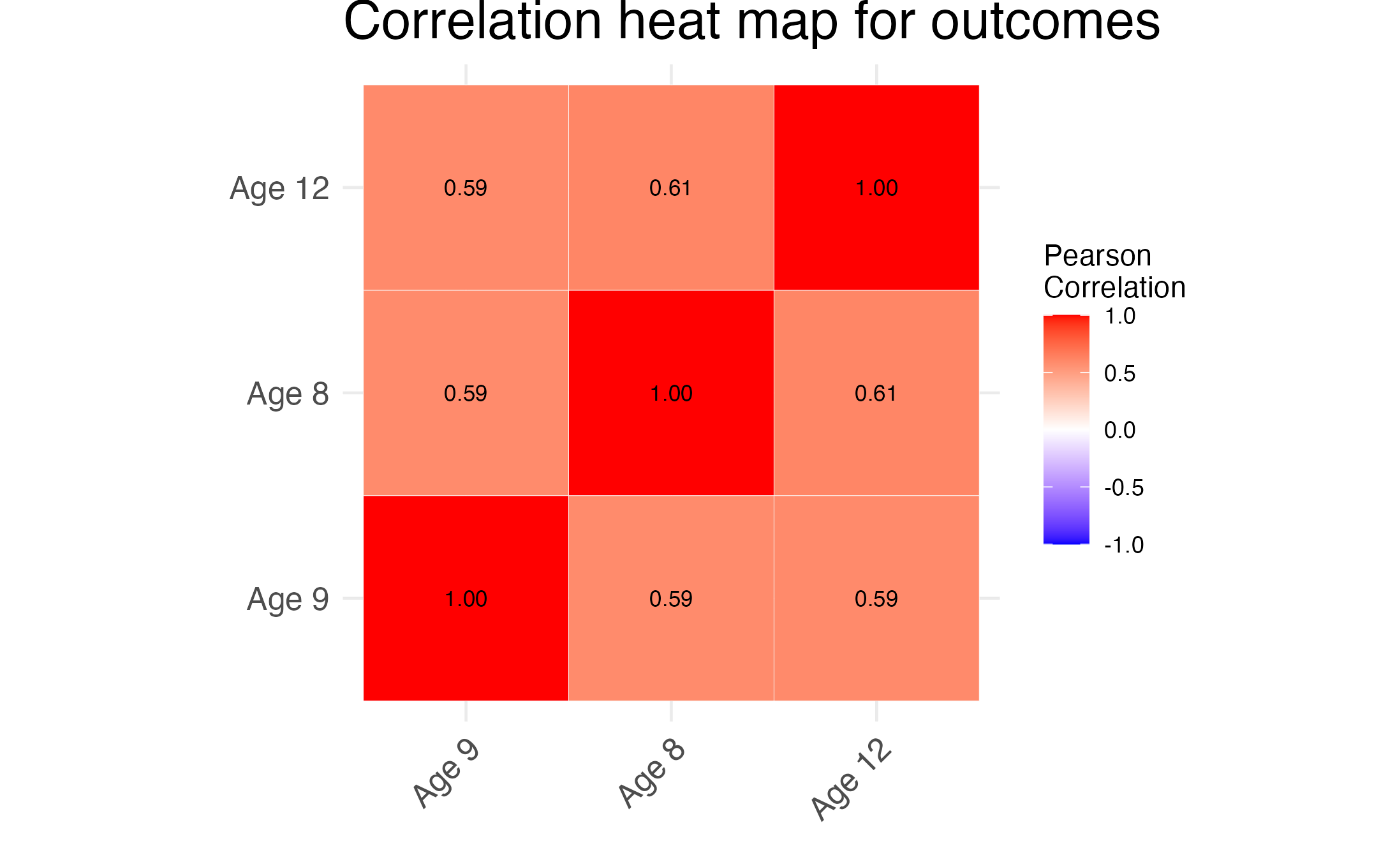


Heat map showing Pearson correlations between the CAST scores reported by parents at different ages, in Environmental Risk (E‐Risk) Longitudinal Twin Study participants with complete post-traumatic stress disorder (PTSD) data and CAST scores for at least one of three time points (N=1,504).

### ****Figure S3****

***Mean scores of parent reported Childhood Autism Spectrum Test (CAST) scores at different ages.***


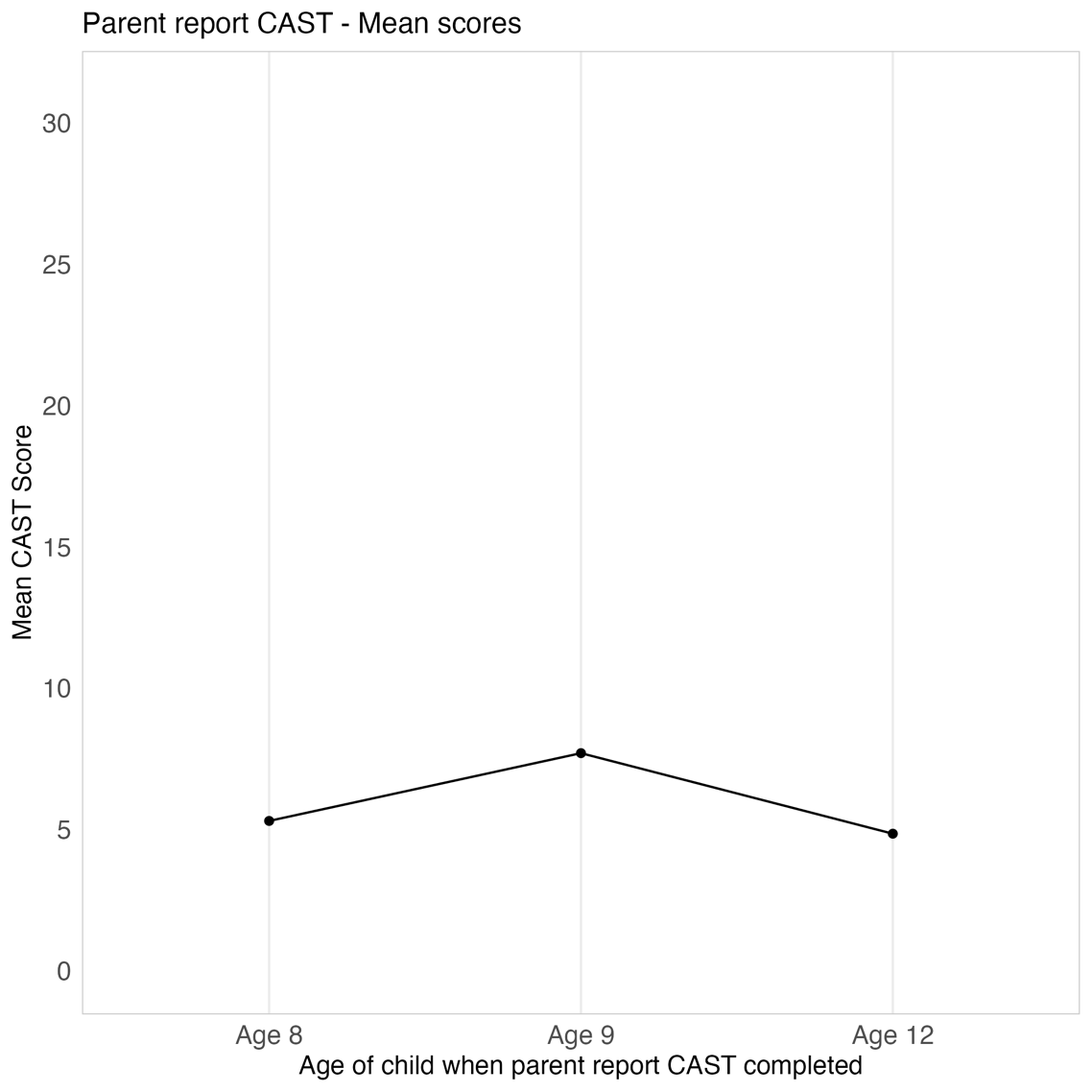


This graph reports the means of the parent reported CAST scores at different ages and shows the stability of the parents’ reports across ages 8, 9 and 12.

### ****Table S1****

***Missingness of parent reported Childhood Autism Spectrum Test (CAST) scores at different ages***

| Age of child when parent reported CAST completed | Missing CAST data in analytical sample with CAST data at age 8, 9 or 12, and complete PTSD data (N=1504) |
| --- | --- |
| Age 8 | 291 (19.4%) |
| Age 9 | 564 (37.5%) |
| Age 12 | 367 (24.4%) |

## Appendix S3: Missing data

### Missing autistic trait data

Table S1 shows data for key outcome measures and demographic variables (including potential confounds) for the analytical sample used in this study where those with missing CAST data at ages 8, 9 and 12 had been removed, and those in the E-Risk sample who had complete PTSD data.

### Table S2

***E-Risk participants with and without missing autistic trait data***

|  | Subset with CAST data at age 8, 9 or 12, and complete PTSD data (analytical sample) (N=1504) | E-Risk sample with complete PTSD data (N=2061) |
| --- | --- | --- |
| Female | 809 (53.8%) | 1082 (52.5%) |
| Family SES |  |  |
| High SES | 596 (38.3%) | 689 (33.4%) |
| Medium SES | 500 (33.2%) | 683 (33.1%) |
| Low SES | 428 (28.5%) | 689 (33.4%) |
| IQ, Mean (SD) | 102 (14.9) | 100 (15.1) |

Data are n/N (%) unless otherwise stated. ‘IQ’ = intelligence quotient; ‘SD’ = standard deviation; ‘SES’ = socio-economic status’.

## Appendix S4: Dimensional measures of psychopathology within the E-Risk cohort at age 18

### Assessment of symptoms of mental health conditions

At age 18, participants were assessed in private interviews about past-year symptoms of mental health conditions (Schaefer et al., 2018). Five externalising-spectrum disorder symptoms were assessed: Diagnostic and Statistical Manual of Mental Disorders 4^th^ edition (DSM–IV) (American Psychiatric Association, 1994) symptoms of alcohol dependence and cannabis dependence assessed via the Diagnostic Interview Schedule (DIS) (Robins et al., 1995); conduct disorder assessed by inquiring about DSM–IV symptoms; symptoms of tobacco dependence assessed via the Fagerstrom Test for Nicotine Dependence (Heatherton et al., 1991); and attention-deficit/ hyperactivity disorder (ADHD) assessed by inquiring about DSM 5^th^ edition (DSM–5) symptoms (Agnew-Blais et al., 2016; American Psychiatric Association, 2013). Four internalising-spectrum disorder symptoms were assessed: DSM–IV symptoms of depression, generalised anxiety disorder, and posttraumatic stress disorder (PTSD) assessed via the DIS (Robins et al., 1995), and symptoms of eating disorder assessed via the SCOFF (Morgan et al., 1999). Thought disorder symptoms were assessed in two ways: first, participants were asked 7 items about delusions and hallucinations (psychotic-like experiences: e.g., “Have other people ever read your thoughts?”; “Have you ever thought you were being followed or spied on?”; “Have you ever heard voices that other people cannot hear?”) (Polanczyk et al., 2010). Second, participants were asked 6 items about unusual thoughts and feelings (prodromal symptoms: e.g., “My thinking is unusual or frightening”; “People or places I know seem different”), drawing on item pools since formalised in prodromal psychosis instruments, including the PRIME-screen and SIPS (Loewy et al., 2011).

### The structure of psychopathology

Using confirmatory factor analysis, two standard models (Brunner et al., 2012; Rindskopf & Rose, 1988) that are frequently used to examine hierarchically structured constructs were estimated: a correlated-factors model with three factors (representing Internalising, Externalising, and Thought Disorder symptoms) and a bi-factor model specifying a General Psychopathology factor (Figure S3) in addition to the three specific factors. Decisions about symptom-factor loadings were guided by the Hierarchical Taxonomy of Psychopathology consortium (<https://medicine.stonybrookmedicine.edu/HITOP/AboutHiTOP>) (Kotov et al., 2017). Symptoms corresponding to disorders of distress (depression, generalised anxiety disorder, and PTSD) and eating pathology loaded on the Internalising factor; symptoms corresponding to disorders of substance use (alcohol, cannabis, tobacco) and oppositional behaviour (conduct disorder) and ADHD loaded on the Externalising factor; and symptoms corresponding to disorders associated with psychosis loaded on the Thought Disorder factor. Confirmatory factor analyses were run as two-level clustered models to account for the nesting of twins within families, with analyses performed in MPlus v7.4 (Muthén & Muthén, 2017) using the robust maximum likelihood estimator (MLR) to provide standard errors that are robust to non-normality and non-independence of observations.

Both models fit the data well as assessed by the Akaike Information Criterion (AIC), Bayesian Information Criterion (BIC) and the Sample Adjusted BIC, although the bi-factor model demonstrated marginally superior fit.

For the correlated-factors model, AIC=42987.116, BIC=43488.486, Sample Adjusted BIC=43205.726. Loadings on each of the three factors were all positive, generally high (all p’s < .001) and averaged 0.680 (Externalising: average loading=0.638; Internalising: average loading=0.654; Thought Disorder: average loading=0.836). Correlations between the three factors were all positive and ranged from 0.552 between Externalising and Thought Disorder to 0.756 between Internalising and Thought Disorder. Thus, this model confirmed that three correlated factors (i.e., Internalising, Externalising, and Thought Disorder) explained the structure of the 11 symptom scales examined in the E-Risk twins at age 18.

For the bi-factor model, AIC=42897.350, BIC=43443.787, Sample Adjusted BIC=43135.609. Loadings on the General Psychopathology factor (“p”) were all positive, generally high (all p’s < .001) and averaged 0.519; the highest standardised loadings were for psychotic symptoms (0.759 and 0.592), major depressive episode (0.718), eating disorders (0.574), and generalised anxiety disorder (0.567). Similarly, the loadings for the three style factors were all positive and averaged 0.507 for Externalising, 0.270 for Internalising, and 0.496 for Thought Disorder. Thus, this model confirmed that a bi-factor structure (i.e., with a General Psychopathology factor and three specific Internalising, Externalising, and Thought Disorder factors) explained the structure of the 11 symptom scales examined in the E-Risk twins at age 18.

### ****Figure S3****

***The structure of psychopathology at age 18 years in the E-Risk Cohort***


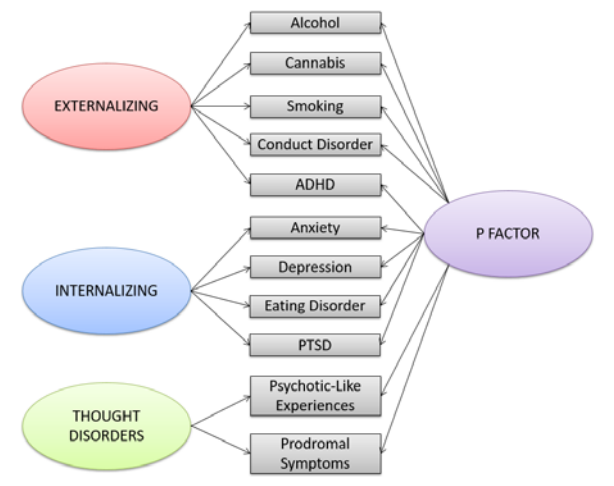
**(**A)


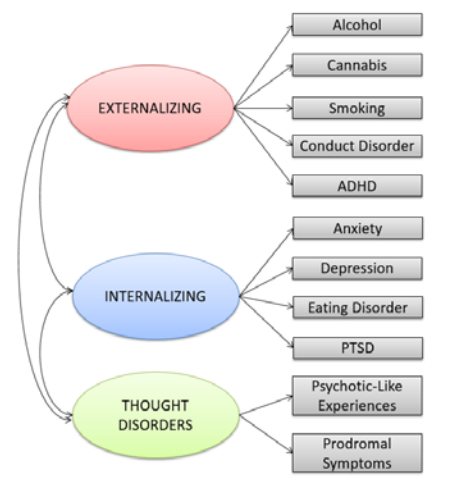
**(**B)

*Note.* (A) Bi-factor model, (B) Correlated-factors model. Coloured ovals represent latent (unobserved) continuous symptom trait factors; grey boxes represent age-18 observed scores on symptom scales corresponding to each disorder. ADHD = attention-deficit/hyperactivity disorder, PTSD = post-traumatic stress disorder. P-Factor represents the factor of General Psychopathology. Figure reproduced from Schaefer et al. (2018).

## Appendix S5: Associations between variables

### Multicollinearity in predictor, confounding and outcome variables

Figure S4 shows that none of the variables included in the analyses breached the multicollinearity threshold (r>0.9).

### ****Figure S4****

**
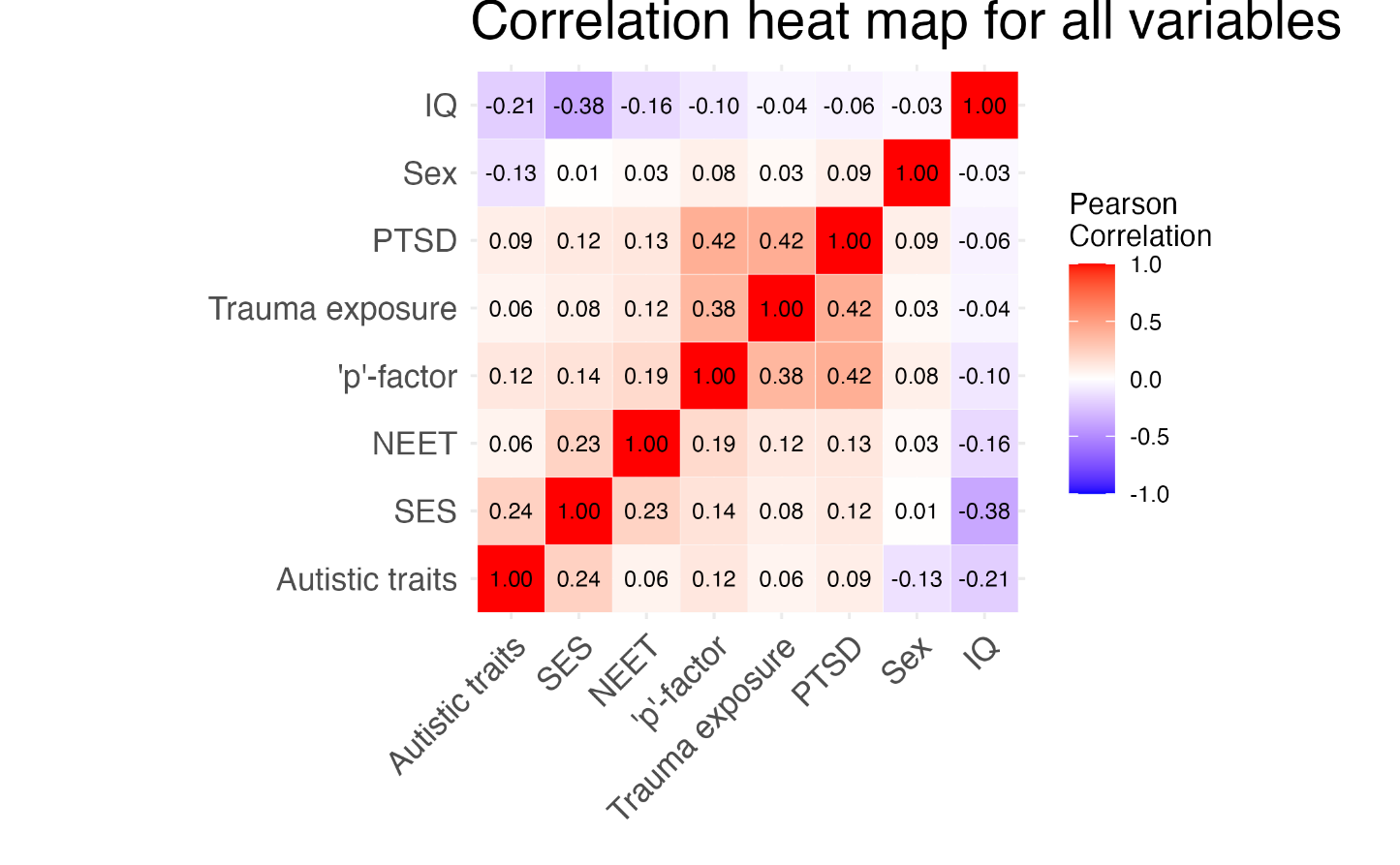
*Pearson correlations between all variables in analytical sample***

Heat map showing Pearson correlations between all variables entered into the analyses.

### Associations between potential confounders and autistic traits

The association between potential confounders and the predictor variable, autistic traits, was examined in the overall sample and trauma-exposed subsample. In the overall sample, children with higher autistic traits were less likely to be female (OR=-0.46, 95% CI=-0.64;-0.27), but were more likely to have lower IQ (Beta=-5.48, 95% CI=-6.80;-4.16). There was no significant association with being in the low SES group (OR=0.91, 95% CI=0.66;1.16). In the trauma-exposed subsample, children with higher autistic traits were more likely to have lower IQ (Beta=-4.95, 95% CI=-7.33;-2.56). There were no significant links between higher autistic traits in children and being female (OR=0.73, 95% CI=0.37;1.09) or being in the low SES group (OR=0.96, 95% CI=0.52;1.40).

## Appendix S6: Sensitivity analyses

### Sensitivity analysis: Using CAST score data at age 8 only

The full analysis was re-run in a sample of participants with CAST data at age 8 (n=1213). The estimates followed similar patterns for unadjusted and adjusted analyses to those yielded in the analyses where missing data was replaced with CAST scores at age 9 and 12. There were marginal differences, as outlined below and indicated in Table S3 for the overall sample (n=1213) and Table S4 in the trauma-exposed subsample (n=369). While the results fell just below conventional levels of statistical significance, the estimates were similar to those obtained in the main analyses potentially reflecting the smaller sample size.

In the overall sample, with trauma exposure as the outcome, the results differed for the unadjusted analysis, with findings just below statistical significance (**OR =** 1.21, 95% CI = 0.97 - 1.51), compared to a statistically significant result in the main analysis (**OR =**1.26, 95% CI = 1.03 - 1.54). Additionally, when controlled for sex (**OR =** 1.24, 95% CI = 0.99 - 1.55) or IQ (**OR =**1.19, 95% CI = 0.95 - 1.49) the relationship between autistic traits and trauma was not significant, whereas this was statistically significant in the main analysis (adjusted for sex (**OR =** 1.29, 95% CI = 1.05 - 1.58) and IQ (**OR =** 1.23, 95% CI = 1.00 - 1.50).

In the trauma-exposed subsample (N=369), the only finding that differed in the sensitivity analysis was the fully adjusted model where PTSD was the outcome which was not statistically significant (**OR =** 1.73, 95% CI = 0.98 - 3.04), compared to the statistically significant finding in the main analysis (**OR =** 1.62, 95% CI = 1.04 - 2.53).

### ****Table S3****

***Sensitivity analysis in those with CAST score data at age 8 only (N=1213).***

| Panel A: Associations with trauma exposure in the overall sample-OR [95% CI] | | | | | |
| --- | --- | --- | --- | --- | --- |
|  | Univariate models | Multivariate models adjusted for: | | | |
|  |  | **Sex** | **IQ** | **SES** | **Sex, IQ and SES** |
| Autistic traits | 1.21 [0.97 - 1.51]* | 1.24 [0.99 - 1.55]* | 1.19 [0.95 - 1.49]* | 1.13 [0.90 - 1.42] | 1.15 [0.91 - 1.46] |
| Female sex | 1.10 [0.86 - 1.40] | *1.14 [0.89 - 1.47]* |  |  | *1.14 [0.88 - 1.47]* |
| IQ | 1.00 [0.99 - 1.00] |  | *1.00 [0.99 - 1.00]* |  | *1.00 [0.99 - 1.01]* |
| Medium SES | 1.15 [0.86 - 1.54] |  |  | *1.13 [0.84 - 1.52]* | *1.13 [0.83 - 1.54]** |
| Low SES | **1.53 [1.13 - 2.07]** |  |  | ***1.47 [1.08 - 2.01]*** | ***1.47 [1.05 - 2.05]**** |
| Panel B: Associations with PTSD diagnosis in the overall sample-OR [95% CI] | | | | | |
|  | Univariate models | Multivariate models adjusted for: | | | |
|  |  | **Sex** | **IQ** | **SES** | **Sex, IQ and SES** |
| Autistic traits | **2.00 [1.25 - 3.20]** | **2.29 [1.40 - 3.76]** | **1.80 [1.13 - 2.87]** | **1.59 [1.01 - 2.51]** | **1.81 [1.11 - 2.95]** |
| Female sex | **2.09 [1.30 - 3.35]** | ***2.43 [1.48 - 3.98]*** |  |  | ***2.45 [1.48 - 4.04]*** |
| IQ | 0.98 [0.97 - 1.00] |  | *0.99 [0.97 - 1.00]* |  | *1.00 [0.98 - 1.01]* |
| Medium SES | **2.11 [1.14 - 3.93]** |  |  | ***1.99 [1.06 - 3.72]*** | ***2.02 [1.07 - 3.82]*** |
| Low SES | **3.84 [2.13 - 6.92]** |  |  | ***3.29 [1.81 - 6.00]*** | ***3.22 [1.69 - 6.13]*** |
| Panel C: Associations with NEET status in the overall sample – OR [95% CI] | | | | | |
|  | Univariate models | Multivariate models adjusted for: | | | |
|  |  | **Sex** | **IQ** | **SES** | **Sex, IQ and SES** |
| Autistic traits | 1.48 [0.98 - 2.24] | 1.52 [0.99 - 2.33]* | 1.15 [0.78 - 1.68] | 0.98 [0.65 - 1.47] | 0.90 [0.60 - 1.35] |
| Female sex | 1.09 [0.74 - 1.59] | *1.17 [0.79 - 1.74]* |  |  | *1.12 [0.74 - 1.71]* |
| IQ | 0.96 [0.95 - 0.97] |  | *0.96 [0.95 - 0.98]* |  | *0.98 [0.96 - 0.99]* |
| Medium SES | 1.69 [0.90 - 3.16] |  |  | *1.70 [0.90 - 3.19]* | *1.41 [0.74 - 2.68]* |
| Low SES | **8.10 [4.73 - 13.87]** |  |  | ***8.17 [4.71 - 14.15]*** | ***6.19 [3.51 - 10.91]*** |
| Panel D: Associations with the ‘p’-factor in the overall sample – Beta [95% CI] | | | | | |
|  | Univariate models | Multivariate models adjusted for: | | | |
|  |  | **Sex** | **IQ** | **SES** | **Sex, IQ and SES** |
| Autistic traits | **3.16 [1.63 - 4.70]** | **3.73 [2.20 - 5.25]** | **2.65 [1.14 - 4.17]** | **2.41 [0.85 - 3.96]** | **2.78 [1.24 - 4.31]** |
| Female sex | **3.12 [1.50 - 4.74]** | ***3.82 [2.20 - 5.43]*** |  |  | ***3.73 [2.12 - 5.34]*** |
| IQ | **-0.12 [-0.18 - -0.06]** |  | ***-0.10 [-0.16 - -0.04]*** |  | *-0.06 [-0.12 - -0.01]* |
| Medium SES | **2.24 [0.41 - 4.07]** |  |  | ***1.89 [0.04 - 3.74]*** | *1.45 [-0.42 - 3.32]* |
| Low SES | **5.13 [3.03 - 7.22]** |  |  | ***4.30 [2.15 - 6.45]*** | ***3.47 [1.23 - 5.71]*** |

Values in **bold text** indicate statistically significant results (p<0.05). Values in *Italic text* are contributions of confounding variables to association between autistic traits and outcomes of interest. All models are adjusted for the non-independence of twin observations. ‘PTSD’ = post-traumatic stress disorder; ‘NEET’ = not in education, employment or training; ‘’p’-factor’ = measure of general psychopathology; ‘IQ’ = intelligence quotient; ‘SES’ = socio-economic status’; ‘OR’ = odds ratio; ‘Beta’ = beta coefficient; ‘95% CI’ = 95% confidence intervals. An astrix indiciates where sensitivity analyses results’ significance differed to those in the main analyses.

### ****Table S4****

***Sensitivity analysis in trauma-exposed young people with CAST score data at age 8 only (n=369)***

| Panel A: Associations with trauma exposure in the trauma-exposed sub-sample-OR [95% CI] | | | | | |
| --- | --- | --- | --- | --- | --- |
| Overall sample: PTSD | Univariate models | Multivariate models adjusted for: | | | |
|  |  | **Sex** | **IQ** | **SES** | **Sex, IQ and SES** |
| Autistic traits | **1.94 [1.15 - 3.26]** | **2.17 [1.24 - 3.78]** | **1.78 [1.07 - 2.96]** | **1.58 [0.93 - 2.65]** | 1.73 [0.98 - 3.04]* |
| Female sex | **2.22 [1.32 - 3.72]** | ***2.46 [1.43 - 4.22]*** | |  | ***2.57 [1.47 - 4.49]*** |
| IQ | 0.98 [0.96 - 1.00] |  | *0.99 [0.97 - 1.00]* |  | *0.99 [0.97 - 1.01]* |
| Medium SES | **2.10 [1.08 - 4.09]** |  |  | ***2.02 [1.02 - 3.96]*** | ***2.07 [1.02 - 4.20]*** |
| Low SES | **3.41 [1.79 - 6.49]** |  |  | ***2.93 [1.51 - 5.66]*** | ***2.91 [1.43 - 5.92]*** |
| Panel B: Associations with NEET status in the trauma-exposed sub-sample – OR [95% CI] | | | | | |
| Overall sample: NEET | Univariate models | Multivariate models adjusted for: | | | |
|  |  | **Sex** | **IQ** | **SES** | **Sex, IQ and SES** |
| Autistic traits | 1.17 [0.62 - 2.21] | 1.17 [0.61 - 2.23] | 0.96 [0.55 - 1.69] | 0.71 [0.36 - 1.41] | 0.63 [0.33 - 1.19] |
| Female sex | 0.94 [0.53 - 1.66] | *0.95 [0.53 - 1.73]* | |  | *0.92 [0.49 - 1.72]* |
| IQ | 0.96 [0.94 - 0.98] |  | *0.96 [0.94 - 0.98]* |  | *0.97 [0.94 - 0.99]* |
| Medium SES | 1.26 [0.49 - 3.25] |  |  | *1.31 [0.50 - 3.40]* | *1.02 [0.38 - 2.76]* |
| Low SES | **6.37 [2.90 - 14.00]** |  |  | ***7.26 [3.14 - 16.82****]* | ***5.68 [2.41 - 13.35]*** |
| Panel C: Associations with the ‘p’-factor in the trauma-exposed sub-sample – Beta [95% CI] | | | | | |
| Overall sample: P factor | Univariate models | Multivariate models adjusted for: | | | |
|  |  | **Sex** | **IQ** | **SES** | **Sex, IQ and SES** |
| Autistic traits | **3.83 [0.66 - 7.00]** | **4.30 [1.20 - 7.39]** | 3.12 [-0.01 - 6.24] | 2.80 [-0.42 - 6.01] | 2.92 [-0.18 - 6.01] |
| Female sex | **5.02 [1.81 - 8.24]** | ***5.46 [2.29 - 8.64]*** | |  | ***5.33 [2.16 - 8.50]*** |
| IQ | **-0.19 [-0.30 - -0.08]** |  | ***-0.17 [-0.28 - -0.06]*** |  | *-0.14 [-0.26 - -0.02]* |
| Medium SES | 1.75 [-2.10 - 5.61] |  |  | *1.47 [-2.40 - 5.35]* | *0.72 [-3.08 - 4.52]* |
| Low SES | **5.84 [1.98 - 9.71]** |  |  | ***4.83 [0.85 - 8.80]*** | ***3.58 [-0.50 - 7.67]*** |

Values in **bold text** indicate statistically significant results (p<0.05). Values in *Italic text* are contributions of confounding variables to association between autistic traits and outcomes of interest. All models are adjusted for the non-independence of twin observations. ‘PTSD’ = post-traumatic stress disorder; ‘NEET’ = not in education, employment or training; ‘’p’-factor’ = measure of general psychopathology; ‘IQ’ = intelligence quotient; ‘SES’ = socio-economic status’; ‘OR’ = odds ratio; ‘Beta’ = beta coefficient; ‘95% CI’ = 95% confidence intervals. An astrix indiciates where sensitivity analyses results’ significance differed to those in the main analyses.

### Sensitivity analysis: Using PTSD within 12 months of assessment as outcome measure

**The main analysis used lifetime PTSD as the PTSD outcome. In this sensitivity analysis, in the full analytical sample (N=1504), we repeated analyses where PTSD was the outcome using PTSD within 12 months of the assessment as the outcome variable. This significantly reduced the number of PTSD cases within sample (N= 63). Findings are shown for the overall sample in Table S5 and trauma-exposed sub-sample in Table S6.**

**The relationships between autistic traits and PTSD in the overall sample were unchanged in this sensitivity analysis. Key differences in the sensitivity analysis findings in the overall sample were that being female was significantly associated with PTSD (OR =1.94, 95% CI = 1.13 - 3.33), which was not significant in the main analysis (female sex OR = 1.16, 95% CI = 0.93 - 1.44). Having medium family SES was also associated with PTSD (OR =1.94, 95% CI = 1.13 - 3.33) in the sensitivity analysis, but this was not significant in the main analysis (OR =1.21, 95% CI = 0.93 - 1.58).**

**In the trauma-exposed sub-sample, the key differences in the sensitivity analysis compared to the main results were that autistic traits were not significantly associated with PTSD when controlling for SES (OR =**1.41, 95% CI = 0.86 - 2.32) nor in the fully adjusted analysis (**OR =**1.54, 95% CI = 0.93 - 2.55). In the main analyses, there was a significant association between autistic traits and PTSD in both of these models; controlling for SES (**OR =** 1.52, 95% CI = 1.00 - 2.32) and the fully adjusted analysis (**OR =**1.62, 95% CI = 1.04 - 2.53).

### ****Table S5****

***Sensitivity analysis in overall sample using PTSD in last 12 months rather than lifetime PTSD.***

| Associations with PTSD diagnosis (<12 months) in the overall sample-OR [95% CI] | | | | | |
| --- | --- | --- | --- | --- | --- |
|  | Univariate models | Multivariate models adjusted for: | | | |
|  |  | **Sex** | **IQ** | **SES** | **Sex, IQ and SES** |
| Autistic traits | **2.02 [1.26 - 3.26]** | **2.21 [1.37 - 3.58]** | **1.90 [1.19 - 3.05]** | 1.53 [0.95 - 2.46] | **1.75 [1.07 - 2.87]** |
| Female sex | **1.94 [1.13 - 3.33]*** | ***2.17 [1.26 - 3.72]*** |  |  | ***2.12 [1.22 - 3.71]**** |
| IQ | 0.99 [0.97 - 1.00] |  | *0.99 [0.98 - 1.01]* |  | *1.01 [0.99 - 1.03]* |
| Medium SES | **2.76 [1.25 - 6.10]*** |  |  | ***2.60 [1.16 - 5.79]**** | ***2.79 [1.24 - 6.29]*** |
| Low SES | **5.44 [2.58 - 11.48]** |  |  | ***4.73 [2.16 - 10.35]*** | ***5.02 [2.20 - 11.45]*** |

Values in **bold text** indicate statistically significant results (p<0.05). Values in *Italic text* are contributions of confounding variables to association between autistic traits and outcomes of interest. All models are adjusted for the non-independence of twin observations. ‘PTSD’ = post-traumatic stress disorder; ‘IQ’ = intelligence quotient; ‘SES’ = socio-economic status’; ‘OR’ = odds ratio; ‘95% CI’ = 95% confidence intervals. An astrix indiciates where sensitivity analyses results’ significance differed to those in the main analyses.

### ****Table S6****

***Sensitivity analysis in trauma-exposed subsample using PTSD in last 12 months rather than lifetime PTSD.***

| Associations with PTSD diagnosis (<12 months) in the trauma-exposed sub-sample-OR [95% CI] | | | | | |
| --- | --- | --- | --- | --- | --- |
|  | Univariate models | Multivariate models adjusted for: | | | |
|  |  | **Sex** | **IQ** | **SES** | **Sex, IQ and SES** |
| Autistic traits | **1.79 [1.09 - 2.94]** | **1.88 [1.14 - 3.09]** | **1.71 [1.05 - 2.80]** | 1.41 [0.86 - 2.32]* | 1.54 [0.93 - 2.55]* |
| Female sex | **1.85 [1.05 - 3.26]** | ***1.96 [1.11 - 3.45]*** |  |  | ***2.01 [1.10 - 3.66]*** |
| IQ | 0.99 [0.97 - 1.01] |  | *0.99 [0.97 - 1.01]* |  | *1.01 [0.98 - 1.03]* |
| Medium SES | **2.56 [1.13 - 5.83]** |  |  | ***2.47 [1.08 - 5.66]*** | ***2.63 [1.13 - 6.11]*** |
| Low SES | **4.50 [2.06 - 9.79]** |  |  | ***4.03 [1.78 - 9.09]*** | ***4.30 [1.85 - 10.01]*** |

Values in **bold text** indicate statistically significant results (p<0.05). Values in *Italic text* are contributions of confounding variables to association between autistic traits and outcomes of interest. All models are adjusted for the non-independence of twin observations. ‘PTSD’ = post-traumatic stress disorder; ‘IQ’ = intelligence quotient; ‘SES’ = socio-economic status’; ‘OR’ = odds ratio; ‘95% CI’ = 95% confidence intervals. An astrix indiciates where sensitivity analyses results’ significance differed to those in the main analyses.

## References

Agnew-Blais, J. C., Polanczyk, G. V., Danese, A., Wertz, J., Moffitt, T. E., & Arseneault, L. (2016). Evaluation of the Persistence, Remission, and Emergence of Attention-Deficit/Hyperactivity Disorder in Young Adulthood. *JAMA Psychiatry*, *73*(7), 713–720. https://doi.org/10.1001/jamapsychiatry.2016.0465

American Psychiatric Association. (1994). *Diagnostic and statistical manual of mental disorders, 4th ed* (pp. xxvii, 886). American Psychiatric Publishing, Inc.

American Psychiatric Association. (2013). *Diagnostic and Statistical Manual of Mental Disorders (DSM-5®)* (5th ed.). American Psychiatric Publishing, Inc.

Brunner, M., Nagy, G., & Wilhelm, O. (2012). A tutorial on hierarchically structured constructs. *Journal of Personality*, *80*(4), 796–846. https://doi.org/10.1111/j.1467-6494.2011.00749.x

Heatherton, T. F., Kozlowski, L. T., Frecker, R. C., & Fagerström, K. O. (1991). The Fagerström Test for Nicotine Dependence: A revision of the Fagerström Tolerance Questionnaire. *British Journal of Addiction*, *86*(9), 1119–1127. https://doi.org/10.1111/j.1360-0443.1991.tb01879.x

Kotov, R., Krueger, R. F., Watson, D., Achenbach, T. M., Althoff, R. R., Bagby, R. M., Brown, T. A., Carpenter, W. T., Caspi, A., Clark, L. A., Eaton, N. R., Forbes, M. K., Forbush, K. T., Goldberg, D., Hasin, D., Hyman, S. E., Ivanova, M. Y., Lynam, D. R., Markon, K., … Zimmerman, M. (2017). The Hierarchical Taxonomy of Psychopathology (HiTOP): A dimensional alternative to traditional nosologies. *Journal of Abnormal Psychology*, *126*(4), 454–477. https://doi.org/10.1037/abn0000258

Loewy, R. L., Pearson, R., Vinogradov, S., Bearden, C. E., & Cannon, T. D. (2011). Psychosis risk screening with the Prodromal Questionnaire—Brief version (PQ-B). *Schizophrenia Research*, *129*(1), 42–46. https://doi.org/10.1016/j.schres.2011.03.029

Moffitt, T. E. & E-Risk Study Team. (2002). Teen-aged mothers in contemporary Britain. *Journal of Child Psychology and Psychiatry, and Allied Disciplines*, *43*(6), 727–742. https://doi.org/10.1111/1469-7610.00082

Morgan, J. F., Reid, F., & Lacey, J. H. (1999). The SCOFF questionnaire: Assessment of a new screening tool for eating disorders. *BMJ (Clinical Research Ed.)*, *319*(7223), 1467–1468. https://doi.org/10.1136/bmj.319.7223.1467

Muthén, L., & Muthén, B. (2017). *MPlus User’s Guide* (8th ed.). Muthén & Muthén.

Odgers, C. L., Caspi, A., Bates, C. J., Sampson, R. J., & Moffitt, T. E. (2012). Systematic social observation of children’s neighborhoods using Google Street View: A reliable and cost-effective method. *Journal of Child Psychology and Psychiatry, and Allied Disciplines*, *53*(10), 1009–1017. https://doi.org/10.1111/j.1469-7610.2012.02565.x

Polanczyk, G., Moffitt, T. E., Arseneault, L., Cannon, M., Ambler, A., Keefe, R. S. E., Houts, R., Odgers, C. L., & Caspi, A. (2010). Etiological and clinical features of childhood psychotic symptoms: Results from a birth cohort. *Archives of General Psychiatry*, *67*(4), 328–338. https://doi.org/10.1001/archgenpsychiatry.2010.14

Rindskopf, D., & Rose, T. (1988). Some Theory and Applications of Confirmatory Second-Order Factor Analysis. *Multivariate Behavioral Research*, *23*(1), 51–67. https://doi.org/10.1207/s15327906mbr2301_3

Robins L, Cottler L, Bucholz K, Compton W. Diagnostic Interview Schedule for DSM-IV. St. Louis: Washington University School of Medicine; 1995.

Schaefer, J. D., Moffitt, T. E., Arseneault, L., Danese, A., Fisher, H. L., Houts, R., Sheridan, M. A., Wertz, J., & Caspi, A. (2018). Adolescent Victimization and Early-Adult Psychopathology: Approaching Causal Inference Using a Longitudinal Twin Study to Rule Out Noncausal Explanations. *Clinical Psychological Science: A Journal of the Association for Psychological Science*, *6*(3), 352–371. https://doi.org/10.1177/2167702617741381

Trouton, A., Spinath, F. M., & Plomin, R. (2002). Twins early development study (TEDS): A multivariate, longitudinal genetic investigation of language, cognition and behavior problems in childhood. *Twin Research: The Official Journal of the International Society for Twin Studies*, *5*(5), 444–448. https://doi.org/10.1375/136905202320906255
